# Reactive CaCO_3_ Formation from CO_2_ and Methanolic Ca(OH)_2_ Dispersions: Transient Methoxide Salts, Carbonate Esters and Sol–Gels

**DOI:** 10.1021/acsphyschemau.4c00041

**Published:** 2024-07-23

**Authors:** Thokozile A. Kathyola, Elizabeth A. Willneff, Colin J. Willis, Peter J. Dowding, Sven L. M. Schroeder

**Affiliations:** † School of Chemical and Process Engineering, University of Leeds, Leeds LS2 9JT, U.K.; ‡ Diamond Light Source, Harwell UK Science & Innovation Campus, Didcot OX11 0DE, U.K.; § School of Design, University of Leeds, Leeds LS2 9JT, U.K.; ∥ Infineum UK Ltd., Abingdon OX13 6BB, Oxfordshire, U.K.

**Keywords:** calcium carbonate, methanol, reactive crystallization, solvent effects, chemical structure

## Abstract

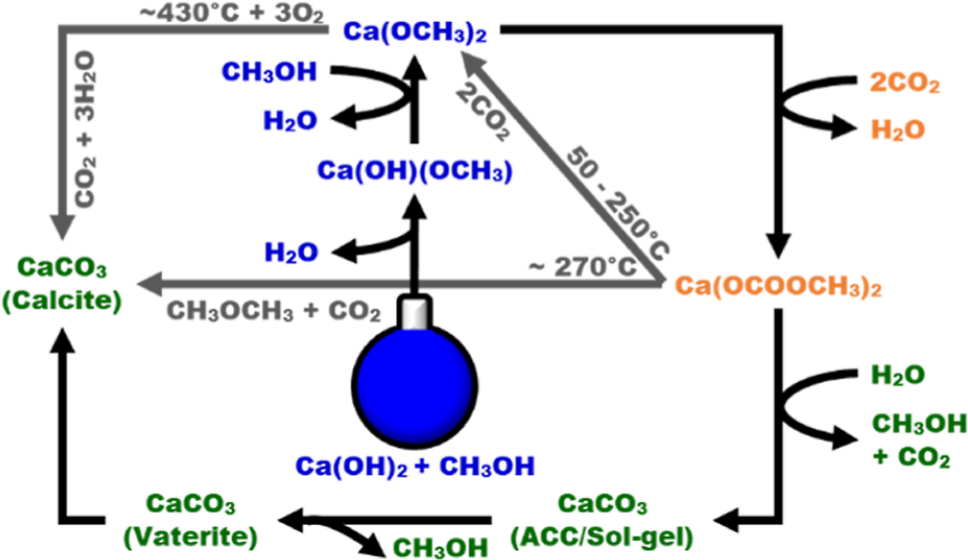

A combination of *ex situ* and *in situ* characterization techniques was used to determine the mechanism of calcium carbonate (CaCO_3_) formation from calcium hydroxide (Ca(OH)_2_) dispersions in methanol/water (CH_3_OH/H_2_O) systems. Mid-infrared (mid-IR) analysis shows that in the absence of carbon dioxide (CO_2_) Ca(OH)_2_ establishes a reaction equilibrium with CH_3_OH, forming calcium hydroxide methoxide (Ca(OH)(OCH_3_)) and calcium methoxide (Ca(OCH_3_)_2_). Combined *ex situ* mid-IR, thermogravimetric analysis (TGA), X-ray diffraction (XRD), X-ray absorption spectroscopy and scanning electron microscopy examination of the reaction product formed in the presence of CO_2_ reveals the formation of calcium dimethylcarbonate (Ca(OCOOCH_3_)_2_). This strongly suggests that carbonation takes place by reaction with the Ca(OCH_3_)_2_ formed from a Ca(OH)_2_ and CH_3_OH reaction. Time-resolved XRD indicates that in the presence of H_2_O the Ca(OCOOCH_3_)_2_ ester releases CH_3_OH and CO_2_, forming ACC, which subsequently transforms into vaterite and then calcite. TGA reveals that thermal decomposition of Ca(OCOOCH_3_)_2_ in the absence of H_2_O mainly leads to the reformation of Ca(OCH_3_)_2_, but this is accompanied by a significant parallel reaction that releases dimethylether (CH_3_OCH_3_) and CO_2_. CaCO_3_ is the final product in both decomposition pathways. For CH_3_OH/H_2_O mixtures containing more than 50 mol % H_2_O, direct formation of calcite from Ca(OH)_2_ becomes the dominant pathway, although the formation of some Ca(OCOOCH_3_)_2_ was still evident in the *in situ* mid-IR spectra of 20 and 40 mol % CH_3_OH systems. In the presence of ≤20 mol % H_2_O, hydrolysis of the ester led to the formation of an ACC sol–gel. In both the 90 and 100 mol % CH_3_OH systems, diffusion-limited ACC → vaterite → calcite transformations were observed. Traces of aragonite were also detected. We believe that this is the first time that these reaction pathways during the carbonation of Ca(OH)_2_ in a methanolic phase have been systematically and experimentally characterized.

## Introduction

Calcium carbonate (CaCO_3_) has been studied extensively due to its abundance in nature and its wide range of economically important applications, e.g. in fuels, pharmaceuticals and construction. CaCO_3_ exists in six different polymorphic forms: three anhydrous crystalline forms–calcite, aragonite and vaterite; two hydrates–monohydrocalcite and ikaite; and amorphous CaCO_3_ (ACC), which exists in both anhydrous and hydrated forms.^1−3^ Despite extensive scientific research, our understanding of what governs CaCO_3_ polymorph selection, polymorphic transformation dynamics, crystallinity and stability is still very limited. The three anhydrous polymorphs have been studied most with a view to their variable morphologies, crystal structures and physicochemical properties. Selectivity toward a particular polymorph of anhydrous CaCO_3_ is known to be determined by variables such as temperature, pH, supersaturation, and the presence of organic additives.^1,2^ However, recent studies have shown that solvents such as alcohols can play a crucial role in determining the polymorphic outcomes or transformations during reactive CaCO_3_ crystallization.^4−19^ For example, the formation and stabilization of ACC, vaterite and aragonite over the thermodynamically favorable calcite has been achieved in solvents with significant alcohol concentrations. The polymorphic outcomes from these reactions have been associated with specific solvent ratios,^8,10,12,13,18,19^ varying solubility of precursors and CaCO_3_ in alcohol/water solvent mixtures,^4,6,7,9,14,18^ retardation of dissolution/recrystallization processes due to alcohol adsorption,^7,9,11,15,16^ and/or formation of alkoxide intermediates.^4,15−17^ Reported relationships between solvent choice and the dominant polymorphic outcome of reactive CaCO_3_ crystallization have not always agreed. This may be due to variations in synthetic methodology, such as reaction duration and the source of Ca^2+^ ions. There is a consensus in that calcite precipitates in alcohol-dominant systems from ACC via metastable vaterite and/or aragonite. However, the mechanism of ACC formation and impact on the product of reactive crystallization of CaCO_3_ is not clear and has only recently become a topic of investigation.

Alkoxides have previously been identified as transient precursors in the formation of ACC by carbonation of alcoholic calcium oxide (CaO) and calcium hydroxide (Ca(OH)_2_) suspensions/dispersions.^4,6,15−17^ However, systematic investigations into the role of alkoxides in CaCO_3_ precipitation have been limited, perhaps because it is generally assumed that CaO and Ca(OH)_2_ do not dissolve in or react with alcohols. However, CaO and Ca(OH)_2_ are slightly soluble in methanol, CH_3_OH, with solubilities of 0.4 and 0.1 g/L respectively.^20^ Furthermore, calcium methoxide, ethoxide and isopropoxide salts have been detected during vaterite synthesis,^6^ stone conversation,^15−17^ cement treatment^21−23^ and flue gas desulfurization,^24^ indicating that reactions between CaO/Ca(OH)_2_ and alcohols to form alkoxides can be significant. These studies also show that the alkoxides convert to ACC followed by vaterite and calcite. In the case of CaO/Ca(OH)_2_ methanolic dispersions, a transient carbonated calcium methoxide complex^25−27^ is formed from calcium hydroxide methoxide, Ca(OH)(OCH_3_), and/or calcium methoxide, Ca(OCH_3_)_2_, prior to the formation of ACC.^21,22,24,28−31^ Three possible routes can therefore be proposed for the precipitation of CaCO_3_ with CO_2_ in methanolic Ca(OH)_2_ systems.

Route I—direct calcium hydroxide carbonation
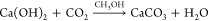
1

Route II—via calcium hydroxide methoxide
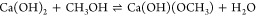
2
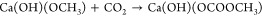
3
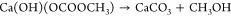
4

Route III—via calcium methoxide
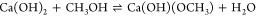
5
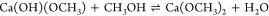
6
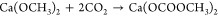
7
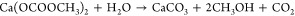
8

Route I is the classic solid–gas reaction of Ca(OH)_2_ and CO_2_ to CaCO_3_, which dominates in the absence of a significant reaction between Ca(OH)_2_ with CH_3_OH or when any formed Ca methoxide reverts back to Ca(OH)_2_ upon reaction with water in the system (this may include water released during methoxide formation).

Routes II and III are multistep reaction sequences that involve the formation and carbonation of the two methoxide salts Ca(OH)(OCH_3_) and Ca(OCH_3_)_2_ to form the equivalent mono- and di-substituted carbonate ester intermediates. These ester intermediates, calcium hydroxide methylcarbonate (Ca(OH)(OCOOCH_3_)) and calcium dimethylcarbonate (Ca(OCOOCH_3_)_2_) have occasionally been reported in the literature, as early as 1926.^25−27^ In systems with low water content (≤20 wt % of H_2_O), these carbonation products tend to form a sol–gel (presumably containing ACC), which can be converted to commercially relevant vaterite/calcite aerogels and xerogels.^25−27^ However, the nature of these carbonate esters have not been resolved, as research has focused on characterizing the CaCO_3_ products.

Here we will for the first time focus on the characterization of the transient carbonate esters formed during the carbonation of Ca(OH)_2_ in CH_3_OH/H_2_O systems. The Ca(OCOOCH_3_)_2_ salt of the carbonic acid methyl ester was comprehensively characterized with a combination of *ex situ* and *in situ* analytical techniques during a study of reaction pathways toward different CaCO_3_ polymorphs from methanolic Ca(OH)_2_ dispersions. Our study shows how the sequential formation of ACC, vaterite and calcite from Ca(OH)_2_ proceeds via the calcium methoxide salts, the carbonate esters and sol–gels. The CH_3_OH content in the CH_3_OH/H_2_O solvent system was varied from 0 to 100 mol % to examine the effects of H_2_O on the rate of precipitation.

## Experimental Section

### Materials

CaCO_3_ was synthesized using Ca(OH)_2_ (94%; L’Hoist), CO_2_ (99.9%; BOC), CH_3_OH (99.9%; Fisher Scientific) and Milli-Q H_2_O. Nitrogen (N_2_; 99.9%; BOC) and helium (He; 99.9%; Air Products) were also used to control the environment during synthesis and X-ray absorption measurements, respectively. Powdered samples of Ca(OCH_3_)_2_ (≥99%—Sigma-Aldrich), calcite (≥99%—Sigma-Aldrich), aragonite, vaterite and ACC were used as references. The aragonite, vaterite and ACC were synthesized using methods proposed by Kitamura et al.,^32^ Shivkumara et al.^33^ and Koga et al.^34^ respectively.

### Calcium Hydroxide Methoxylation

Ca(OH)_2_ was mixed with pure methanol (100 mol %) for 24 h in a 1 L baffled glass reactor, under constant N_2_ flow of 33 mL/min. The temperature and stirring rate were maintained at 28 ± 1 °C and 400 rpm, respectively. The solid product was vacuum filtered before characterization.

### Calcium Carbonate Formation

CaCO_3_ was synthesized for the *ex situ* experiments by carbonating a dispersion of Ca(OH)_2_ (0.05 mol) in 4 mol of pure (100 mol %) and wet (90 mol %) CH_3_OH. CO_2_ was bubbled through the dispersions at a rate of 33 mL/min for a duration of 40 min. Agitation and heating were maintained for 15 min after CO_2_ addition was stopped to allow for complete reaction. The waxy white precipitate product from the 100 mol % system was vacuum filtered before characterization. The 90 mol % product was a sol–gel (Figure S1), so no filtration was required. All experiments were reproducibly carried out using either a 250 mL Quickfit Drechsel bottle or a Radley’s Carousel 6 Plus Reaction Station equipped with six 250 mL round-bottomed flasks. The temperature and stirring rate were maintained at 28 ± 1 °C and 400 rpm, respectively. It was possible to scale up the reactor system to 1 L with no variations in the results. For the *in situ* experiments, dispersions with varying CH_3_OH content (0 to 100 mol %) were prepared by mixing Ca(OH)_2_ (0.53 mol) with 750 mL of solvent. The reactive crystallization processes were carried out in a 1 L baffled glass reactor under a constant N_2_ flow (30 mL/min). The temperature and stirring rate were maintained at 28 ± 1 °C and 400 rpm, respectively. The dispersion was carbonated for a duration of 60 min at a rate of 70 mL/min.

### Mid-Infrared (Mid-IR) Spectroscopy

*Ex situ* Fourier transform mid-IR spectra were collected for all samples using a Thermo Fisher Nicolet 10 iS10 spectrometer equipped with a ZnSe attenuated total reflectance (ATR) crystal. All spectra were an average of 32 scans obtained at a resolution of 4 cm^–1^ from 4000 to 650 cm^–1^. The measurements were collected and processed using the Thermo Fisher OMNIC software. Conversely, *in situ* mid-IR spectra were collected every minute for 75 min using a Bruker Alpha FTIR spectrometer equipped with a Hellman Analytics DPR 210 ZnSe ATR probe. All spectra were an average of 64 scans obtained at a resolution of 4 cm^–1^ from 4000 to 650 cm^–1^. The measurements were collected and processed using the OPUS 7.0 software. All *ex situ* and *in situ* data were analyzed using the Gaussian function in the Fityk 1.3.1 curve fitting software.^35^

### Thermogravimetric Analysis (TGA)/Mid-IR

Thermal analysis was performed using a Mettler Toledo TGA-DSC 3+ analyzer operated at 60 °C min^–1^ from 30 to 650 °C with an N_2_ flow of 50 mL/min to elute evolved gas to the spectrometer. The TGA analyzer was connected to a Thermo Fisher Nicolet 10 iS10 spectrometer equipped with a transmission flow cell. All spectra were an average of 32 scans obtained at a resolution of 4 cm^–1^ from 4000 to 650 cm^–1^. The measurements were collected and processed using the Thermo Fisher OMNIC software.

### X-ray Diffraction (XRD)

XRD was performed using a PANalytical X’Pert-Pro powder X-ray diffractometer with Cu Kα radiation (λ = 1.54056 Å) operated at 40 kV and 40 mA. Samples were placed on a zero-background silicon sample holder and scanned over a 2θ range of 5 to 80° at a scan rate of 0.08° min^–1^ and step size of 0.03°. Scans were collected every 15 min for a duration of 60 to 96 h. XRD data were processed using the PANalytical HighScore Plus software.^36^

### X-ray Absorption Spectroscopy

Total electron yield Ca K-edge X-ray absorption spectra were collected from 4000–4800 eV at Diamond Light Source on beamline B18 with the storage ring operating with an electron current of 300 mA at energy of 3 GeV.^37^ Measurements of the two samples were acquired at room temperature under a constant He environment. All X-ray absorption spectroscopy (XAS) data were processed and analyzed using Athena in the Demeter software package.^38^ Fourier transformed extended X-ray absorption fine structure (EXAFS) were extracted over a k-range from 3 to 8 Å^–1^ with a k-weight of 3. Theoretical EXAFS scattering paths were calculated using ATOMS and FEFF6 in Artemis.^38^ Theoretical Fourier transforms were obtained by fixing the amplitude reduction factor (S_O_^2^) at 0.7 while letting the interatomic distance (*R*), coordination number (*N*_atom_), Debye–Waller factor (σ_2_) and zero-energy correction (Δ*E*_0_) values vary freely for all coordination shells around the central, X-ray absorbing, Ca atom. Experimental EXAFS data were fitted over an *R*-range from 1 to 5 Å with monohydrocalcite^39^ and vaterite (6-layered monoclinic)^40^ model structures.

### Scanning Electron Microscopy (SEM)

The powdered 100 mol % CH_3_OH and reference samples were coated with 15 nm of iridium and analyzed using a Hitachi SU8230 microscope operated at 2.0 kV. Cryo-SEM was used to characterize the 90 mol % CH_3_OH sol–gel product. The gel was frozen in liquid nitrogen, cleaved, coated with platinum, and analyzed using a Thermo Fisher Scientific Helios G4 CX DualBeam microscope operated at 1.0 kV. Energy dispersive X-ray (EDX) spectra were also collected.

## Results and Discussion

### Methoxide Salt Formation in 100 mol % CH_3_OH

#### Mid-IR

**Figure 1:**
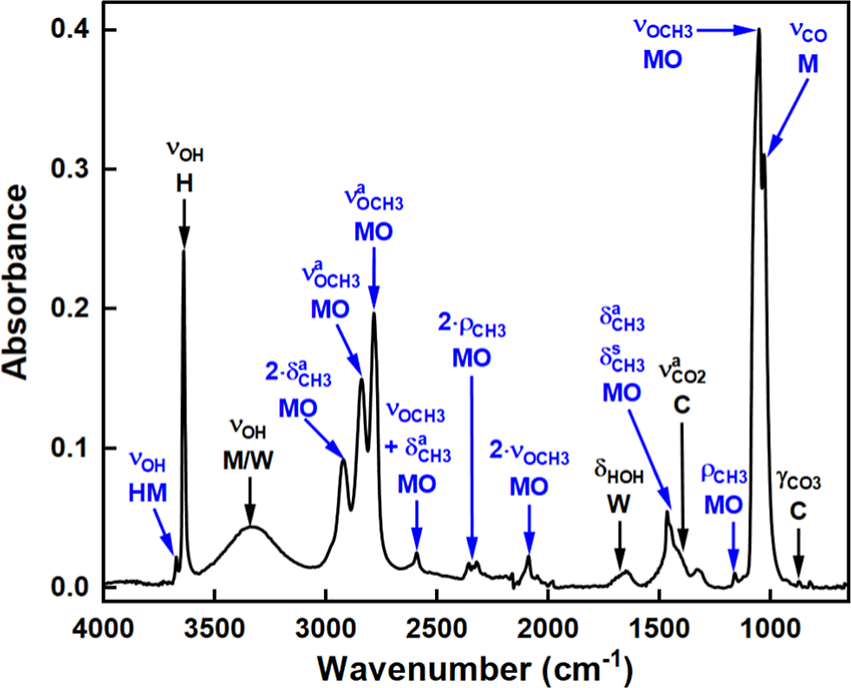
Mid-IR spectrum of the product from the methoxylation of Ca(OH)_2_. Vibrations due to Ca(OH)_2_ (H), calcite (C), CH_3_OH (M), Ca(OH)(OCH_3_)(HM), Ca(OCH_3_)_2_ (MO) and H_2_O (W) are highlighted.

After reacting Ca(OH)_2_ with 100 mol % CH_3_OH for 24 h, vibrational bands characteristic of Ca(OH)_2_ and Ca(OCH_3_)_2_ were identified in the mid-IR spectrum (Figure 1) of the obtained product.^24,29,41^ A sharp OH stretch (ν_OH_) at 3641 cm^–1^ confirmed the presence of unreacted Ca(OH)_2_. A calcite impurity was identified through the asymmetric carbonate stretch (ν_CO_3__^a^—1452 cm^–1^) and out-of-plane bending (γ_CO_3__—874 cm^–1^) vibrations. The formation of the di-substituted methoxide salt is indicated by the relatively intense methoxy stretching (ν_OCH_3__—1053 cm^–1^), asymmetric methyl stretching (ν_CH3_^a^—2841 and 2785 cm^–1^), asymmetric methyl in-plane bending (δ_CH_3__^a^—1467 cm^–1^) and methyl rocking (ρ_CH_3__—1162 cm^–1^) vibrations. Minor 2·δ_CH3_^a^ (2922 cm^–1^) and 2·ν_OCH_3__ (2090 cm^–1^) overtones and a ν_OCH_3__ + δ_CH_3__^a^ combination band (2593 cm^–1^) were also observed.

The remaining peaks were assigned to residual CH_3_OH, with ∼3339 cm^–1^ ν_OH_ and 1031 cm^–1^ ν_CO_ vibrations, and H_2_O formed during methoxylation, with bands at ∼3339 (ν_HOH_) and 1642 (δ_HOH_) cm^–1^. The minor feature at 3674 cm^–1^ was attributed to a ν_OH_ stretch from the mono-substituted methoxide Ca(OH)(OCH_3_)).^24^ Ultimately, the presence of IR vibrations from both the mono- and disubstituted methoxide salts confirms that the Ca(OH)_2_ methoxylation proceeds via Reactions 5 and 6 in route III.^24,28^ The di-substituted methoxide salt (Ca(OCH_3_)_2_) is the main product of the reaction and is reasonably stable in the presence of H_2_O. In previous studies, notably different ratios of the two methoxide salts in the final methoxylation product were reported, with Ca(OH)(OCH_3_) content ranging from ∼92 wt %^28^ to trace amounts.^24^ This variation likely arises because Ca(OCH_3_)_2_ must be kept dry, as it otherwise converts to Ca(OH)_2_ upon reaction with H_2_O—possibly via Ca(OH)(OCH_3_).

### Carbonate Ester Formation in 100 mol % CH_3_OH

#### Mid-IR

**Figure 2:**
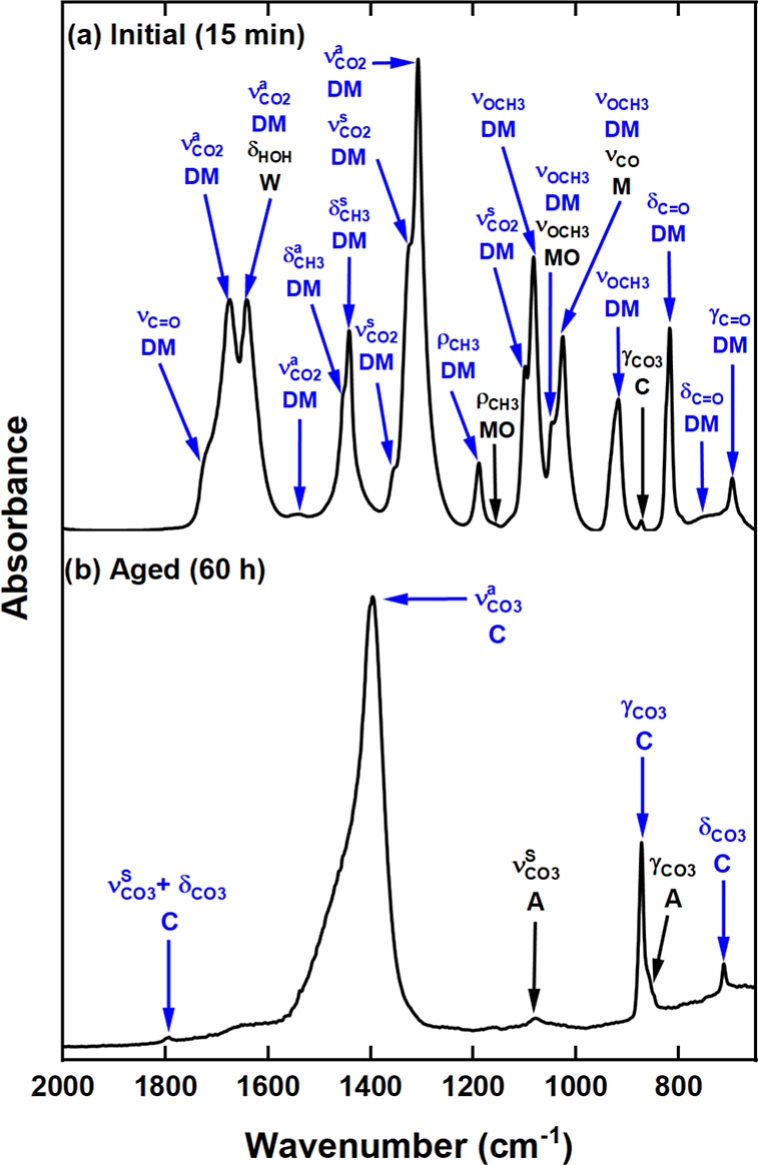
Mid-IR spectra of the (a) initial and (b) aged 100 mol % CH_3_OH postcarbonation product. Vibrations due to CH_3_OH (M), Ca(OCH_3_)_2_ (MO), Ca(OCOOCH_3_)_2_ (DM),H_2_O (W), calcite (C) and aragonite (A) are highlighted.

The carbonation of Ca(OH)_2_ dispersed in 100 mol % CH_3_OH was investigated and yielded a waxy white precipitate as the initial product after ∼15 min. The mid-IR spectrum (Figure 2a) of this product shows multiple vibrations especially in the 1800 to 650 cm^–1^ region. The full deconvoluted mid-IR spectrum (from 4000 to 650 cm^–1^) and all vibrational mode assignments are presented in Figure S2 and Table S1. The precipitation of CaCO_3_ directly from Ca(OH)_2_ via route I (Reaction 1) was excluded by the appearance of multiple vibrations not characteristic of CaCO_3_. In the mid-IR spectra of the six CaCO_3_ polymorphs^14,42−44^ there are at most seven characteristic features due to the carbonate group. The vibrations at ∼1453, 873, and 696 cm^–1^ in Figure 2a may stem from asymmetric stretching (ν_CO_3__^a^), out-of-plane bending (γ_CO_3__) and in-plane-bending (δ_CO_3__) vibrations, respectively. The 873 cm^–1^ feature indicates the presence of trace amounts CaCO_3_ species in the form of either calcite or vaterite. However, the appearance of multiple stronger features in other regions of the IR suggests the presence of an additional carbonated species.

The occurrence of Ca(OH)_2_ methoxylation (Reactions 5 and 6), in conjunction with carbonation, was evidenced by ρ_CH_3__ (1160 cm^–1^) and ν_OCH_3__ (1049 cm^–1^) methoxy vibrations (Figure 2a). The absence of the Ca(OH)(OCH_3_) ν_OH_ stretch at ∼3674 cm^–1^ (identified in Figure 1) alluded to the sole presence of Ca(OCH_3_)_2_. Notably, Ca(OCH_3_)_2_ formed at a faster rate (<40 min; Figure 2a) in the presence of CO_2_ compared to the CO_2_-free system (24 h; Figure 1). This can be attributed to a forward shift in the reaction equilibrium (Reactions 5 and 6) due to (i) a conversion of the methoxide to a carbonated species (Reaction 7); and/or (ii) catalytic activity of carbonic acid (H_2_CO_3_) formed via a reaction of the CO_2_ with an OH^–^ from CH_3_OH and H_2_O. Ultimately, the presence of Ca(OCH_3_)_2_ confirmed that the calcite and aragonite, detected after 60 h of aging (Figure 2b), were precipitated via route III (Reactions 5–8).^25,26^ Hence, the majority of the peaks observed in Figure 2a must be from the Ca(OCOOCH_3_)_2_ ester salt intermediate.

To the best of our knowledge, the mid-IR spectrum in Figure 2a is the first reported for the Ca(OCOOCH_3_)_2_ ester salt. Consequently, the post-carbonation IR assignments presented in this paper (Figure S2 and Table S1) were based on a comparative analysis of various compounds with similar chemical structures. These reference compounds included: calcium acetate monohydrate,^45,46^ calcium propionate monohydrate,^47^ potassium methyl carbonate,^48^ lithium methyl carbonate,^49,50^ magnesium methoxy methyl carbonate,^51^ dimethyl carbonate,^52^ and dimethyl dicarbonate.^53^ Characteristic ester peaks due to the methoxycarbonyl C=O and C(=O)–O stretching were identified (Table S1) at 1717 ± 15 cm^–1^ (ν_C=O_), 1660 ± 25 and 1309 ± 1 cm^–1^ (ν_CO_2__^a^), 1344 ± 21 cm^–1^ (ν_CO_2__^s^), 799 ± 43 cm^–1^ (δ_C=O_), and 694 ± 3 cm^–1^ (γ_C=O_).^54^ The Ca(OCOOCH_3_)_2_ also exhibited methyl/methoxy vibrations at 1449 ± 6 cm^–1^ (δ_CH_3__^a^); 1437 ± 8 cm^–1^ (δ_CH_3__^s^); 1190 cm^–1^ (ρ_CH_3__); and 1022 ± 76 cm^–1^ (ν_OCH_3__), which were absent in the mid-IR spectrum of Ca(OCH_3_)_2_ (Figure 1). Further evaluation of the reference spectra highlighted the possibility of Ca(OCOOCH_3_)_2_ conformational polymorphism, hydration or dimerization. The vibrational bands associated with both cis–cis and cis–trans conformers have been observed in the mid-IR spectra of dimethyl carbonate^52^ and dimethyl dicarbonate.^53^ Conformational variations in the Ca(OCOOCH_3_)_2_ structure can be achieved by varying the orientation of the terminal methyl (−CH_3_) groups (see Figure S3). Hence, it is conceivable that the post-carbonation product from 100 mol % CH_3_OH contains more than one carbonate ester conformer. This is supported by the multiple methoxy ν_OCH_3__ stretching vibrations in the 975 ± 125 cm^–1^ region (Figures 2a and S2). The methoxy (−OCH_3_) group normally accounts for 12 out of 18 vibrational modes of the methoxycarbonyl anion (CH_3_OCO_2_^–^).^48,54^ However, crystallization of Ca(OCOOCH_3_)_2_ reduces the CH_3_OCO_2_^–^ (point group: *m*)^48^ site symmetry, which leads to the removal of double/triple degeneracies and the appearance of IR inactive vibrations. These effects have previously been observed in calcite (32),^55^ aragonite (*m*)^56^ and vaterite (1 + 2)^57^ mid-IR spectra due to the lowering of the CO_3_^2–^ (6*–*2*m*) site symmetry.^58,59^ Moreover, the probabilities of hydration or dimerization, as observed with calcium propionate monohydrate^47^ and lithium methylcarbonate^49,50^ respectively, have not been discounted. Indeed, in Figure S2b also a minor δ_HOH_ contribution at 1642 cm^–1^ is evident, which can be linked to crystallized water.^60^ Ultimately, the mid-IR results confirm the occurrence of the Ca(OCOOCH_3_)_2_ intermediate and its transformation into aragonite and calcite (Figure 2b) via route III (Reactions 5–8). Affirmation of Ca(OCOOCH_3_)_2_ conformational polymorphism, hydration or dimerization through the vibrational data would require an in-depth experimental and ab initio IR and Raman study of the pure ester in both solid and liquid form. Such an ab initio study was beyond the scope of the present work, but could account for multiple additional factors including solute–solvent and solute–solute interactions.

#### TGA-IR

**Figure 3:**
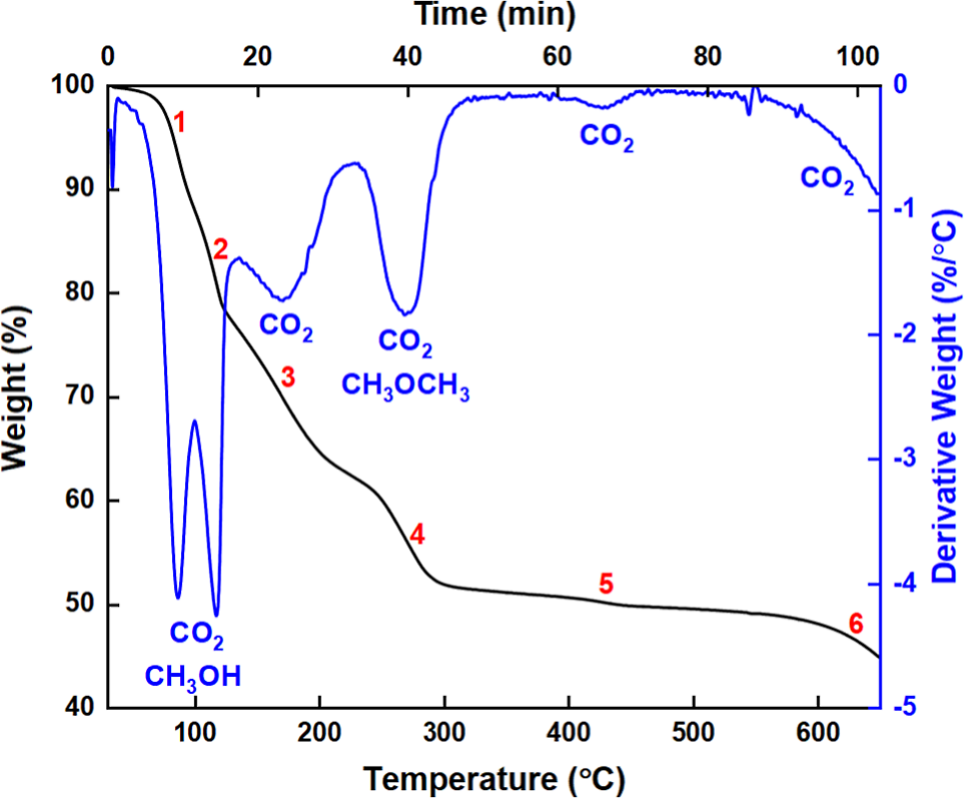
TGA (black) and DTG (blue) plots of the thermal decomposition of Ca(OCOOCH_3_)_2_ into CO_2_, CH_3_OCH_3_, Ca(OCH_3_)_2_ and CaCO_3_. Residual CH_3_OH was also present. The red numbers indicate the six stages of decomposition.

Thermal analysis did not confirm or disprove the hydration of the Ca(OCOOCH_3_)_2_ ester (Figures 3 and S4). The expected mass loss due to H_2_O (at about 100 °C) was obscured by mass losses from CO_2_ and residual CH_3_OH (at 83 and 115 °C). It was also difficult to distinguish between H_2_O and CH_3_OH in mid-IR spectra of the gas phase collected downstream of the TGA (Figure S4a,b) based on the hydroxide stretching (ν_OH_) features at ∼3300 cm^–1^. Like lithium methylcarbonate,^50^ the thermal decomposition of Ca(OCOOCH_3_)_2_ proceeded via two reaction pathways, through
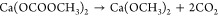
9
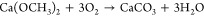
10and
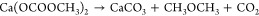
11

The carbonate ester decomposed in four stages between 40 and 380 °C (Figures 3 and S4). The absence of dimethyl ether (CH_3_OCH_3_) at 83, 115, and 169 °C (stage 1 to 3, as indicated by the numbers in red in Figure 3, and data in Figure S4b–d) and a total mass loss of about 23 wt % confirmed Reaction 9 as the main degradation pathway. The Ca(OCH_3_)_2_ reaction product decomposed into CaCO_3_ at about 430 °C (stage 5/Reaction 10). This result agrees with the literature values of 430 and 450 °C for the pure Ca(OCH_3_)_2_ salt.^61,62^Reaction 11 occurred 40 min into the decomposition process, at 269 °C (stage 4 in Figures 3 and S4e), with an expected mass loss of ∼10 wt %, close to the temperature of 252 °C reported for the decomposition of lithium methylcarbonate.^50^ In both cases, the CH_3_OCH_3_ product was discernible from CH_3_OH due to characteristic methyl ether rocking (ρ_CH3_)/stretching (ν_COC_) peaks in the 1200 to 830 cm^–1^ IR region (Figure S4e).^50,54^ Similar thermal studies of organic carbonate esters have previously associated ether formation with the absence of β-hydrogens.^63^ The α-hydrogens present in esters such as dimethyl carbonate and Ca(OCOOCH_3_)_2_ cannot dissociate from the alkyl group, which results in the formation of an ether instead of the respective alcohol (in this case CH_3_OH) and alkene. Hence the CH_3_OH (mass loss of ∼16 wt %) observed at stages 1 and 2 in the TGA can be attributed to incomplete filtration and/or post-filtration drying. Finally, the CaCO_3_ formed during stages 4 and 5 in the TGA began to decompose into CaO from about 583 °C. Collectively, the TGA results show that crystalline CaCO_3_ can also be obtained from Ca(OCOOCH_3_)_2_ via thermal treatment (up to 500 °C), which is an alternative pathway to the hydrolysis reaction in route III (Reaction 8). Notably, calcite has previously been obtained at 280 °C from ACC synthesized by carbonating methanolic and ethanolic dispersions of CaO.^4,5^

#### XRD

**Figure 4:**
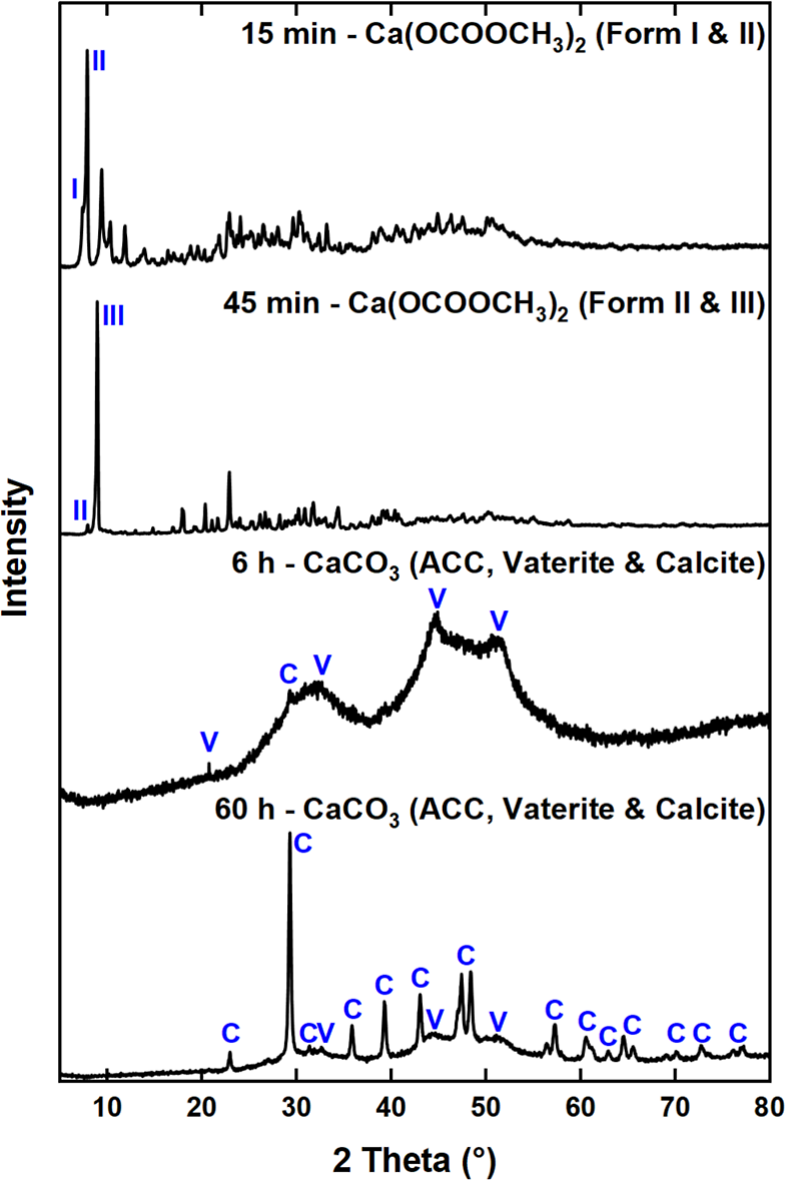
Time-resolved XRD patterns showing the transformation of Ca(OCOOCH_3_)_2_ into ACC, vaterite (V) and calcite (C).

Time-resolved XRD confirmed the presence of Ca(OCOOCH_3_)_2_ and showed its transformation into different forms of CaCO_3_ (Figures 4 and S5). The initial pattern (Form I in Figure S5a) showed multiple features from 6 to 80°, with a very strong peak at 7.32° (*d*-spacing of 12.1 Å). Most of the observed peaks do not fit with diffraction data of Ca(OCH_3_)_2_, vaterite, aragonite and calcite.^40,56,64,65^ The assignment of some minor peaks was inconclusive. Some of these peaks could be attributed to 112, 111, and 104 reflections of vaterite, aragonite and/or calcite. Comparisons with other organic salts revealed that an intense peak between 4 and 8° is characteristic of hydrated carboxylic acid esters.^47^ In the case of calcium propionate monohydrate, a peak occurs at 7.28°, in line with our results, and was attributed to a −200 reflection. This dominant reflection was also apparent in the XRD patterns for hydrate esters of butanoic, pentanoic and hexanoic carboxylic acids.^47^ All four of these carboxylic acid esters have a monoclinic *P*21/*a* space group, perhaps indicating that Ca(OCOOCH_3_)_2_ has a monoclinic structure as well. A definitive assignment of a space group has to await analysis of a single crystalline sample. The XRD patterns (Figure S7a) also indicate that over time the intensity of the form I peak at 7.32° decreased as a weaker peak appeared at 7.82° (*d*-spacing of 11.3 Å) associated with form II. This second peak can transform further into a higher intensity peak at 8.92° (form III; *d*-spacing of 9.91 Å) (Figure 4).

A comparison of the three XRD patterns (Figure S5a) reveals that each has unique reflections, which suggests that they represent different forms of the Ca(OCOOCH_3_)_2_ ester. The observed decrease in lattice spacing was most likely due to hydrolysis of the ester by atmospheric H_2_O (Reaction 8). The possibility that the three forms relate to different polymorphs, as already suggested by the mid-IR (*vide supra*), cannot be excluded. Interestingly, differences in the XRD patterns of initial (∼15 min) post-carbonation products from two experiments (Figure S5a) were due to slight over-carbonation in experiment 2. The increased amount of H_2_CO_3_ most likely catalyzed the conversion of form I to II. After exposure to ambient atmosphere for 60 to 96 h, formation of both vaterite and calcite was evident in both experiments (Figures 4 and S5b). This agrees with the results of mid-IR spectra of the aged post-carbonation sample, which showed the presence of both polymorphs (Figure 2b). The time-resolved XRD showed that the Ca(OCOOCH_3_)_2_ transformed into calcite via metastable ACC and vaterite. Minor contributions from vaterite were present in the XRD patterns of form II and III of the Ca(OCOOCH_3_)_2_ ester and in the two broad diffuse features characteristic of ACC.^66−68^ The kinetics and mechanism of the formation of calcite via ACC and vaterite have previously been explored in various alcohol–water systems.^10,12−17^ It is likely that the formation of the two metastable CaCO_3_ polymorphs is kinetically favored in supersaturated systems in the presence of methanol because both the reactant, Ca(OH)_2_, and the most stable polymorph, calcite, are only weakly soluble in methanol.^12,20,69^

#### XAS

**Figure 5:**
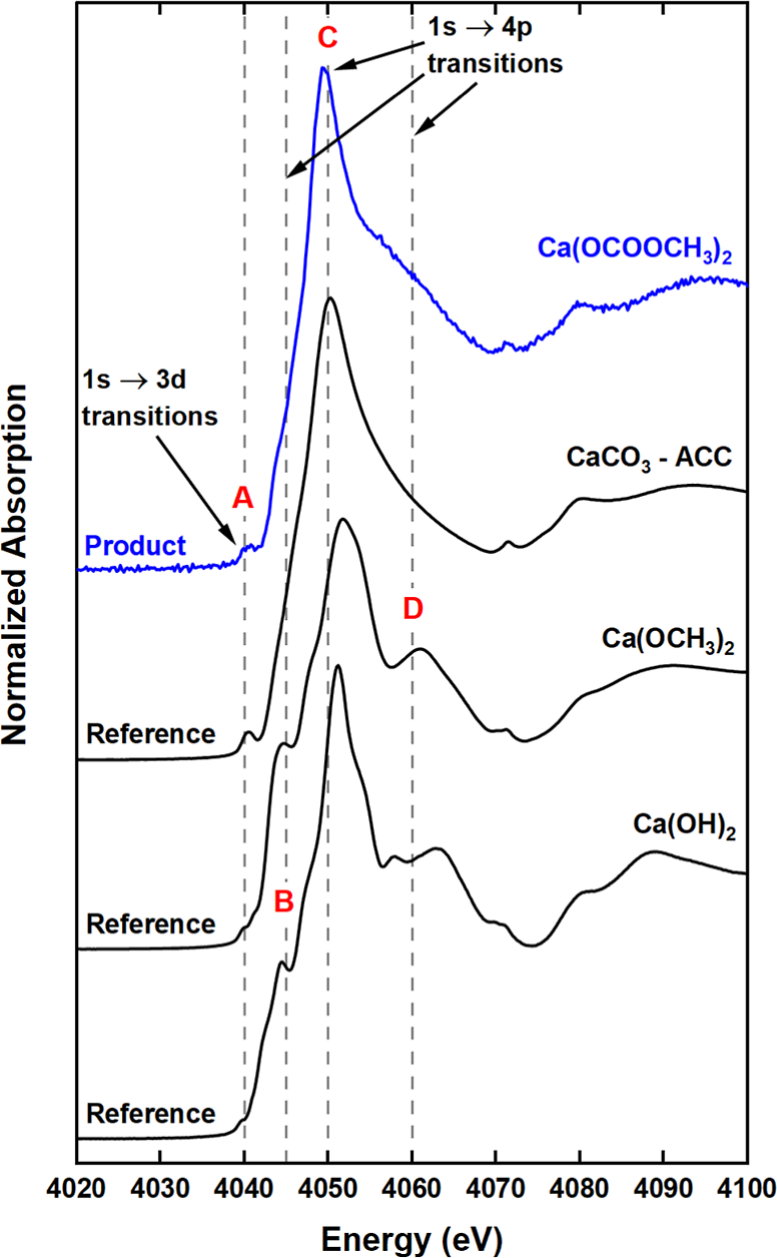
Ca K-edge XANES of Ca(OCOOCH_3_)_2_ compared to Ca(OH)_2_, Ca(OCH_3_)_2_ and ACC standards.

Ca K-edge XAS provided an insight into the electronic structure of Ca(OCOOCH_3_)_2_. The XANES spectrum (Figure 5) showed considerable reductions in features due to 1s → 4p electronic transitions (from 4045 to 4060 eV; labeled B–D), compared to the Ca(OH)_2_ and Ca(OCH_3_)_2_ spectra. These features are, respectively, defined by (B) interactions of Ca 4p with neighboring Ca 3*d*/4s and C π* states;^70−73^ (C) the scattering and coordination number of the first shell neighboring oxygen atoms;^74^ and (D) the orientation/collinearity of the anion.^75,76^ Initially, it was assumed that the post-carbonation product (at the time of the measurement) mainly consisted of ACC due to the similar non-definitive 1s → 4p features and prominent 1s → 3d dipole forbidden transition (at 4040 eV; labeled A). However, a review of the Ca K-edge XANES for hydrated calcium acetate^77,78^ and calcium propionate^79^ revealed analogous spectral features. The two monohydrates exhibit a sharp peak at 4050 eV and a slight modulation at 4060 eV similar to Ca(OCOOCH_3_)_2_ in Figure 5a. The presence of the relatively minor post-edge feature at 4060 eV (labeled D in Figure 5a) has been linked to hydration in calcium acetate monohydrate.^78^ This agrees with the hypothesis made above that the structure of post-carbonation product is most likely Ca(OCOOCH_3_)_2_·*x*H_2_O. However, one also needs to consider the influence of the packing arrangement of OCOOCH_3_^–^ ions on the XANES. Such a structure effect is known for aragonite, where a non-collinear arrangement of the CO_3_^2–^ ions in anhydrous aragonite contributes to the diminution of this 1s → 4p feature (Figure S6).

As has been established for various calcium-containing compounds,^74^ the intensity of peak C at 4050 eV is indicative of the oxygen coordination number (*N*_O_) in the first coordination shell around the Ca^2+^ centers. The intensity indeed increases from vaterite and calcite (*N*_O_ = 6) to aragonite (*N*_O_ = 9) (Figure S6). The spectra in Figure 5a suggested that the first shell N_O_ for the carbonate ester was greater than the 6 for Ca(OH)_2_. This was confirmed by quantitative EXAFS analysis (Figure S7a). Since both the ester and ACC have unknown structures, various CaCO_3_ models were used for the EXAFS fitting. The best models were chosen based on relative residuals (*R*-factor) and reduced chi-square (χ^2^) values.^38,80^ Good fit results for ACC (R-factor of 1.51%) were obtained using a 6-layered monoclinic vaterite model (Figure S7a).^40^ The first Ca–O shell *N*_O_ (5.5) and *R* (2.36 Å) values are similar to those previously reported for synthetic ACC.^81^ The N_O_ suggests that the ACC in this study is anhydrous unlike various other biogenic and synthetic ACC samples with reported N_O_ values of about 8, akin to monohydrocalcite (CaCO_3_·H_2_O).^3,82−84^ Short-range order beyond this first shell is usually not reported for stable/pure ACC samples. However, some studies have shown carbon contributions in the second and third shell.^82,85^*N*_c_ values of 1.5 and 3 have been reported for these two shells at 3.03 and 3.36 Å respectively.^82^ A similar fitting method of varying coordination numbers was applied, which could explain the similarity to the ACC EXAFS results reported in this paper.

Conversely, the Ca(OCOOCH_3_)_2_ ester EXAFS (Figure S7a) showed a preference to the hexagonal CaCO_3_·H_2_O structure^39^ (*R*-factor of 1.11%). Two oxygen environments, with a combined *N*_O_ of about 9, were identified at 2.32 and 2.50 Å. It was initially considered that these oxygens were purely due to the methoxycarbonyl groups, much like the carbonates in aragonite.^86^ However, fitting of the subsequent shells revealed a partiality to oxygens from the water molecules included in the CaCO_3_·H_2_O model. This reiterates the possible presence of crystallized H_2_O, as observed in the mid-IR and XANES. Notably, the EXAFS of the ester was found to be dominated by Ca–O scattering with minimal contribution from the relatively heavy Ca scatterers (at about 4 Å), unlike Ca(OH)_2_ but similar to vaterite. This suggests disorder in the system.

#### SEM

**Figure 6:**
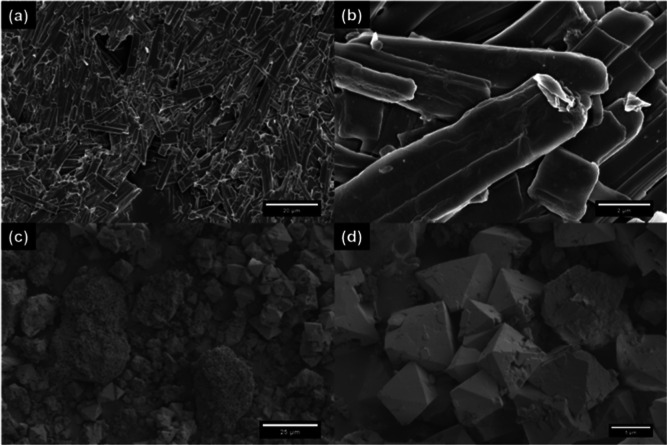
SEM micrographs of (a,b) layered rod-like Ca(OCOOCH_3_)_2_ particles synthesized from the 100 mol % methanolic Ca(OH)_2_ dispersion and (c,d) polyhedral calcite particles obtained from over-carbonating the dispersion.

Finally, the morphology of the Ca(OCOOCH_3_)_2_ precipitate was determined using SEM. Figure 6a shows that the powder consisted of layered polydisperse rod-like particles ranging from 2 to 34 μm. The particles were visibly fused together, explaining the waxy nature of the post-carbonation product. Similar micro-sized Ca(OCOOCH_3_)_2_ needles have previously been reported.^87^ The final rod-like shape is distinctly different from previously determined morphologies of the three crystalline CaCO_3_ polymorphs^2^ and the Ca(OH)_2_ and Ca(OCH_3_)_2_ precursors.^61^ However, it is comparable to the morphology of calcium hexanoate monohydrate particles,^47^ which suggests that the ester may have a monoclinic crystal structure akin to those of the hydrated carboxylic acid esters. It is likely that the ester has a hexagonal substructure akin to vaterite,^88^ considering the EXAFS (Figure S7a) showed a preference to the hexagonal CaCO_3_·H_2_O structure.^39^ A closer inspection of the SEM revealed that end-to-end assembly of Ca(OCOOCH_3_)_2_ nanoparticles most likely led to the formation of the layered rod-like structures. Figure 6b shows what looks like nanoparticles embedded in the layers. EDX analysis (Figure S8) revealed a Ca/C/O ratio of about 19:31:51 which is comparable to the theoretical ratio of 21:25:50 and indicates the chemical structure of the particles is Ca(OCOOCH_3_)_2_ after taking into account carbon impurities and hydrogen contributions.

The carbonation rate was doubled in order to examine the suggestion (*vide supra*) that H_2_CO_3_ formed during carbonation promotes the conversion of Ca(OCOOCH_3_)_2_. A combination of distinct micro-sized polyhedral particles and clusters of mixed nano- and micro-sized particles can clearly be seen in the SEM of the overcarbonated product (Figure 6c,d). Mid-IR confirmed that the polyhedral particles were calcite and the clusters were a mixture of Ca(OCOOCH_3_)_2_ and aragonite. The presence of vaterite was not confirmed as the IR did not show a distinct in-plane bending (δ_CO_3__) vibration at 745 cm^–1^.

### CaCO_3_ Sol–gel Formation in 90 mol % CH_3_OH

#### Mid-IR

**Figure 7:**
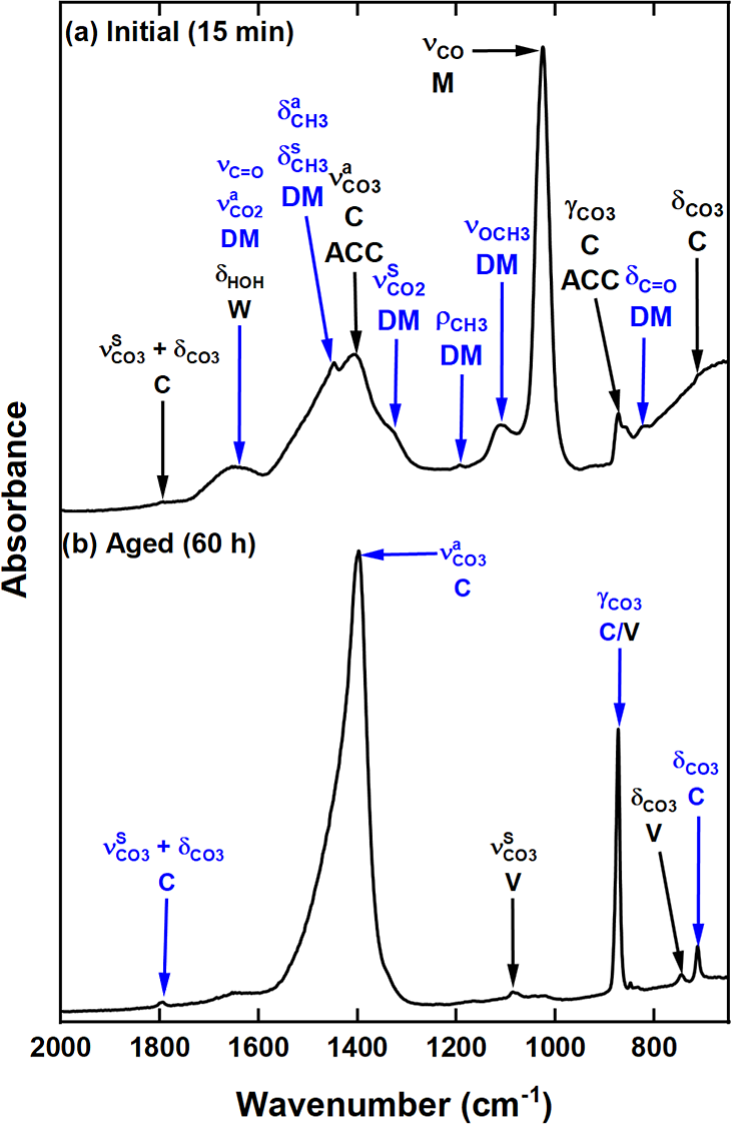
Mid-IR spectra of the (a) initial (sol–gel) and (b) aged (precipitate) 90 mol % CH_3_OH post-carbonation product. Vibrations due to CH_3_OH (M), Ca(OCOOCH_3_)_2_ (DM), calcite (C), vaterite (V), ACC and H_2_O (W) are highlighted.

The formation of CaCO_3_ from the 90 mol % CH_3_OH Ca(OH)_2_ dispersion proceeded via route III (Reactions 5–8), similar to the 100 mol % system. However, the post-carbonation product in the 90 mol % CH_3_OH system was a translucent sol–gel (Figure S1), very different to the waxy precipitate from 100 mol % methanol. Figure 7a shows the mid-IR spectrum of the sol–gel product (15 min) with features associated with CH_3_OH (ν_CO_—1026 cm^–1^), dH_2_O (δ_HOH_—1660 cm^–1^), Ca(OCOOCH_3_)_2_ (ν_C=O_/ν_CO2_^a^—1660 cm^–1^; δ_CH_3__^a^/δ_CH_3__^s^—1450 cm^–1^; ν_CO_2__^s^—1335 cm^–1^; ρ_CH_3__—1192 cm^–1^; ν_OCH_3__—1100 cm^–1^; and δ_C=O_—825 cm^–1^), ACC (γ_CO_3__—860 cm^–1^) and calcite (δ_CO_3__—712 cm^–1^).

The presence of calcite in the sol–gel indicates an increased conversion/CaCO_3_ precipitation rate relative to the Ca(OCOOCH_3_)_2_ product (Figure 2a), due to the presence of the H_2_O. This would promote H_2_CO_3_ formation and Ca(OH)_2_ solubility, where the concentration of Ca^2+^ ions doubles from 0.04 to 0.08 g/L (Figure S9). H_2_CO_3_ promotes the hydrolysis of Ca(OCOOCH_3_)_2_ (Reaction 8), which was also observed in the XRD (Figure 4) and SEM (Figure 6c) of overcarbonated 100 mol % CH_3_OH dispersions. The mid-IR spectrum (Figure 7b) of the aged (60 h) post-carbonation product confirmed that both calcite and vaterite (δ_CO_3__—745 cm^–1^) were formed via ACC in line with results for the 100% CH_3_OH system (Figure 2b).

#### XRD

**Figure 8:**
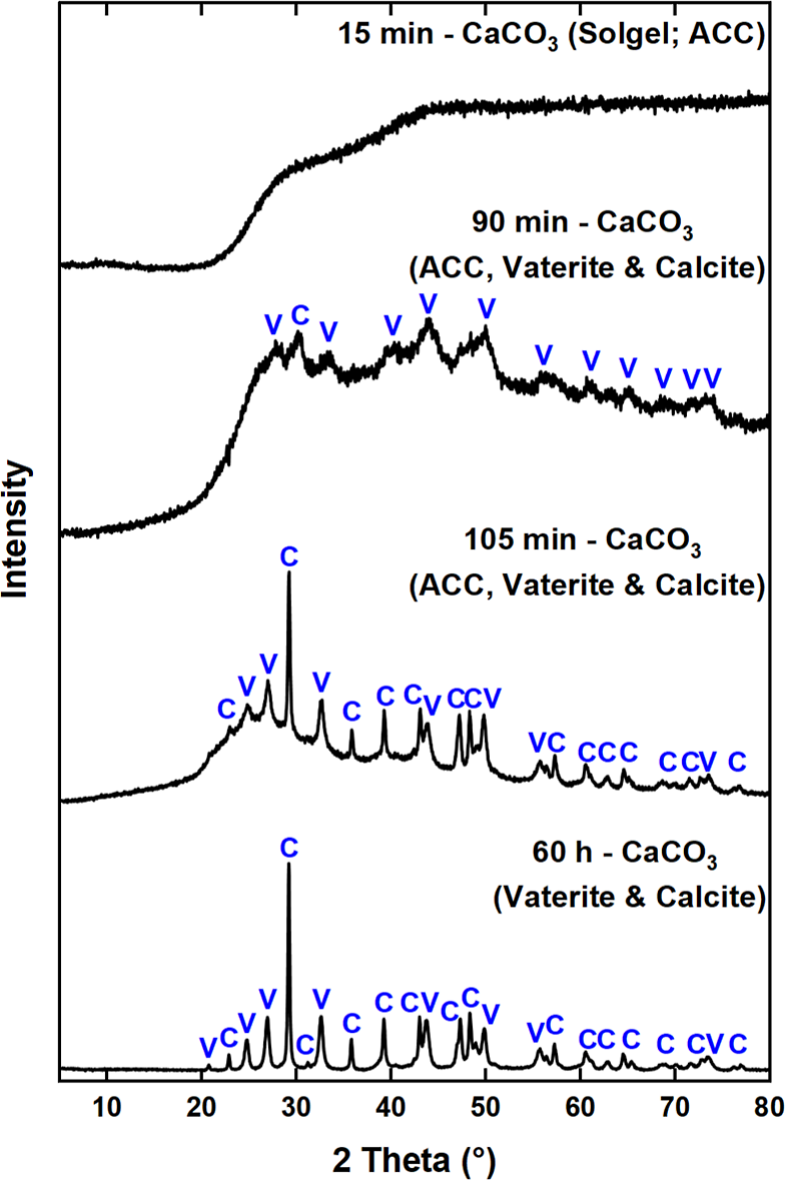
Time-resolved XRD patterns showing the transformation of the ACC sol–gel into vaterite (V) and calcite (C).

The transformation from ACC to vaterite to calcite was also confirmed by time-resolved XRD (Figure 8). Broad diffuse maxima were evident in the initial XRD pattern, confirming the amorphous nature of the sol–gel. Vaterite peaks became more apparent after ∼90 min as the sol–gel underwent syneresis (i.e., gel shrinkage/CH_3_OH expulsion). Beyond 105 min the post-carbonation product consisted mainly of vaterite and calcite. Evidently, CaCO_3_ precipitated at a faster rate in the presence of 10 mol %H_2_O than in the pure system (Figures 4b and S7), which only had trace amounts of vaterite even after 6 h. Notably, the gelation of carbonated methanolic dispersions has previously been observed.^4,25−27^

This paper represents the first time the sol–gel has been characterized, rather than the resulting aerogel or xerogel. The formation of the metastable ACC and vaterite polymorphs from the gel system can be attributed to limited ion diffusion.^89^ The highly supersaturated environment created by the viscous gel media is akin to the “solvent cages” created by the relatively less polar ethanol and isopropanol.^12,90^ In this case, the reaction of Ca(OH)_2_ and CH_3_OH precludes the significant influence of such molecular interactions at the facets of the hydroxide. These interactions are reported to inhibit the precipitation of the thermodynamically stable calcite and aragonite.^12^

#### XAS

**Figure 9:**
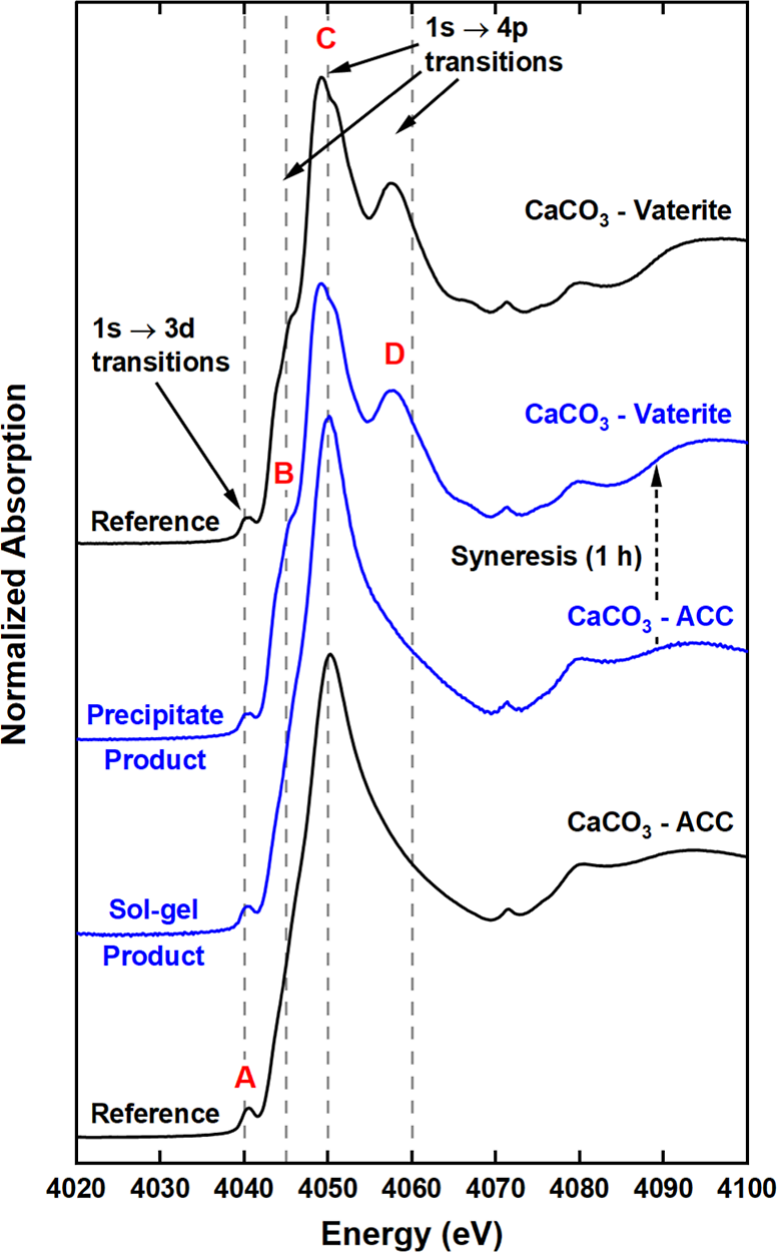
Ca K-edge XANES of the ACC sol–gel (15 min) and dried precipitate (1 h) compared to ACC and vaterite standards.

The absence of distinct 1s → 4p features at 4045 (B) and 4060 (D) eV in the sol–gel Ca K-edge XANES spectra (Figure 9a) were indicative of ACC. This was supported by similarities with the ACC reference XANES (including the position of feature C) and the lack of order beyond the first O coordination shell (at 2.41 Å) in the complementary EXAFS data (Figure 9b). However, fitting of the EXAFS using the CaCO_3_·H_2_O model^39^ showed that the gel was more similar to the Ca(OCOOCH_3_)_2_ than ACC (Figure S7a) with an N_O_ value of about 9. This is most likely due to the methanol in the gel. After gel syneresis (∼1 h post-carbonation) the 1s → 4p XANES features (B and D in Figure 9a) became more pronounced due to crystallization. A comparison with the XANES spectra of the three crystalline CaCO_3_ polymorphs (Figure S6) confirmed the presence of vaterite in the precipitate (Figure S1c). This is confirmed by a good EXAFS fit with an R-factor of 1.45%, which was obtained using the vaterite crystal structure.^40^

#### SEM

**Figure 10:**
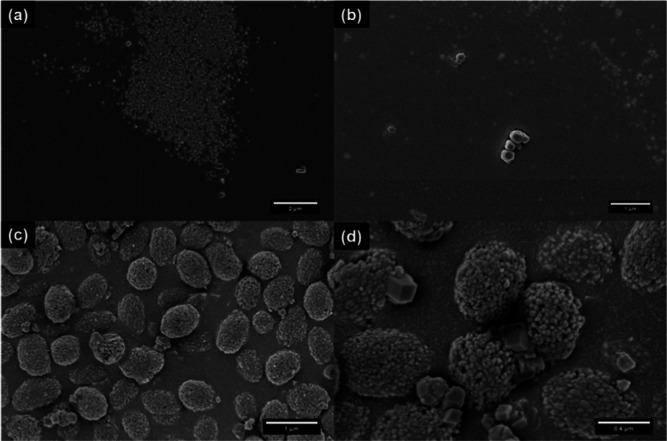
(a,b) Cryo-SEM of the sol–gel post-carbonation product from the 90 mol % methanolic dispersion showing clusters of spherical ACC particles; and (c,d) SEM of the product after drying showing vaterite spherules and traces of rhombohedral calcite particles.

Cryo-SEM (Figure 10a) showed that the sol–gel was composed of a smooth continuous phase containing clusters of 110 ± 30 nm spherical ACC particles. Figure 10b shows traces of crystalline aggregates, which could be the calcite that was detected in the mid-IR (Figure 7a). EDX analysis of the gel showed that Ca only accounts for about 0.2 wt % compared to the 33 and 67 wt % due to C and O respectively. The Ca content was significantly less than the expected amount for both Ca(OCOOCH_3_)_2_ (21 wt %) and CaCO_3_ (40 wt %). This can be attributed to hydrolysis and condensation reactions, which result in the formation of a sol–gel with 96 wt % CH_3_OH/H_2_O and 4 wt % of CaCO_3_. Notably, gelation most likely occurred via a three-step diffusion-limited aggregation route i.e. (i) hydrolysis of the Ca(OCOOCH_3_)_2_ ester to form ACC nanoparticles (Reaction 8); (ii) aggregation of the ACC nanoparticles; and (iii) condensation of the system to form the gel.^2,26,89^ The hydrolysis and condensation reactions can be catalyzed by either the acid (H_2_CO_3_) and/or the base (Ca(OH)_2_) in the system.^91^ The spherical nature of the ACC nanoparticles observed in Figure 10a suggests that the hydrolysis and condensation occur in a high pH system.^91,92^ Gelation in this 90 mol % CH_3_OH system can also occur due to the simultaneous hydrolysis and condensation of the Ca(OCH_3_)_2_ or non-hydrolytic elimination of the Ca(OCOOCH_3_)_2_ ester.^91,93,94^ The latter involves a reaction of the ester with Ca(OCH_3_)_2_ or CH_3_OH to form −Ca–O–Ca-bonds. As can be seen in Figure S1c, further condensation of the gel led to syneresis and the subsequent precipitation of CaCO_3_.^93^ The SEM micrographs of the precipitate (Figure 10c,d) showed that an hour after carbonation the product mainly consisted of vaterite framboids. The 730 ± 90 nm framboids consisted of 40 ± 10 nm spherical vaterite particles. The vaterite particles were notably half the size of the ACC precursors (∼110 nm) present in the initial sol–gel (Figure 10a). This decrease in size has previously been observed for the transformation of ACC to crystalline vaterite via a dissolution–precipitation process.^16,95,96^ Further inspection of the SEM images (Figure 10d) also showed the growth of rhombohedral calcite particles. The observed sequence of polymorphic transformations, from ACC to vaterite to calcite, agrees with the previously discussed results of both the 90 and 100 mol % systems and literature electron microscopy studies on nonclassical crystal growth of CaCO_3_.^16,97^

### Influence of H_2_O on Gelation/Precipitation

**Figure 11:**
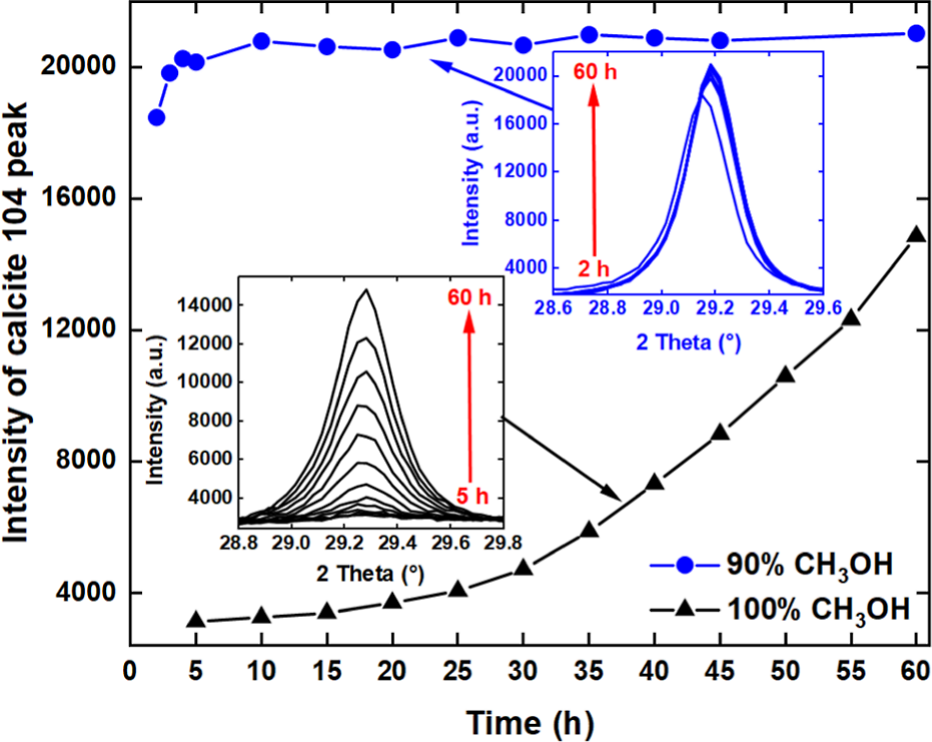
The precipitation of calcite (based on its 104 XRD reflection at ∼29°) from Ca(OH)_2_ as a function of time in the 90 and 100 mol % CH_3_OH systems.

**Figure 12:**
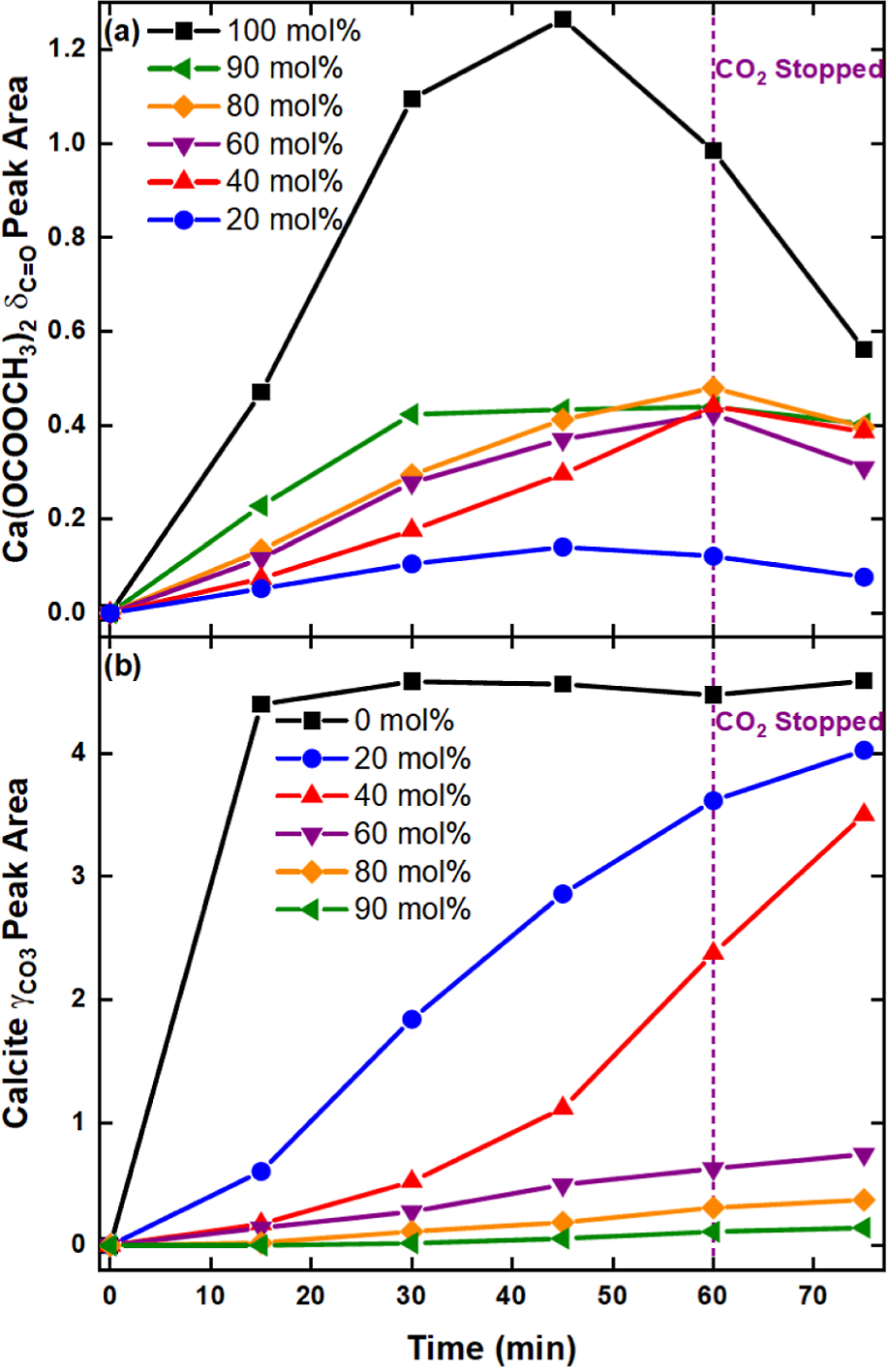
The precipitation of (a) calcite (based on the γ_CO_3__ IR peak at ∼873 cm^–1^) and (b) Ca(OCOOCH_3_)_2_ (based on the δ_C=O_ IR peak at ∼820 cm^–1^) as a function of time from the 0 to 100% CH_3_OH systems.

Comparison of the products obtained from the 90 and 100 mol % CH_3_OH highlighted that H_2_O increased the rate of CaCO_3_ precipitation. This was mostly evident through the strong 104 calcite reflection at about 29° in the time-resolved XRD data (Figures 4 and 8). Figure 11 shows that the maximum amount of calcite was obtained within 10 h from the sol–gel (diluted system). Conversely, only 70% of this maximum was achieved after 60 h of Ca(OCOOCH_3_)_2_ (pure system) hydrolysis by atmospheric H_2_O. A systematic *in situ* mid-IR study (Figure S10) was carried out to further determine the effects of H_2_O on gelation and Ca(OCOOCH_3_)_2_/CaCO_3_ precipitation. Seven Ca(OH)_2_ dispersions with varying amounts of CH_3_OH (0 to 100 mol %) were investigated. As expected, only Ca(OCOOCH_3_)_2_ was detected in the 100 mol % CH_3_OH system (Figure S10a). There was an observable reduction in the intensity and resolution/number of to the ester IR peaks compared to the *ex situ* mid-IR (Figure 2a). The variations were mostly apparent in the 1800 to 1300 cm^–1^ region, where the strong methoxycarbonyl (ν_CO_2__) and methyl (δ_CH_3__) vibrations appear. Additionally, there was no evidence of the previously detected Ca(OCH_3_)_2_ precursor. These observations can be attributed to the relatively large solvent volume (750 mL), which can sometimes mask solute IR vibrations. Mid-IR is known to be very sensitive to contributions from solvents such as H_2_O and CH_3_OH.^98^ For example, the Ca(OCH_3_)_2_ ν_OCH_3__ vibration at 1049 cm^–1^ has been masked by the strong CH_3_OH ν_CO_ at 1026 cm^–1^. Quantitative analysis of the Ca(OCOOCH_3_)_2_ δ_C=O_ peak at about 820 cm^–1^ (Figure 12a) showed that the optimal amount of the ester was obtained 45 min into carbonation. Beyond this point the Ca(OCOOCH_3_)_2_ decreased as it began to hydrolyze into CaCO_3_ (Reaction 8). Hydrolysis was also evident in other systems after carbonation was stopped. Noticeably, an increase in H_2_O/H_2_CO_3_ resulted in a faster esterification/precipitation rate in the 40 and 60 mol % systems compared to the 80 mol %. The maximum amount of Ca(OCOOCH_3_)_2_ obtained in these two systems was comparable to the 90 mol %, but the stability of the ester increased with decreasing H_2_O.

Figure S10 and Table S2 show that above the 50 mol % threshold, CH_3_OH dictates the reaction pathway by which CaCO_3_ is formed. This correlates with the formation of Ca(OCOOCH_3_)_2_ and gelation up to about 60 mol %. A similar trend in gelation and precipitation has been reported in relation to the formation of the ester intermediate.^25^ ACC was confirmed as the dominant polymorph (γ_CO_3__—863 cm^–1^) in the initial post-carbonation product obtained from the 60, 80, and 90 mol % CH_3_OH dispersions (Figure S10b–d).

Sol–gel formation occurred in all three cases, whereby gelation rate increased with H_2_O but the gel stability decreased. Conversely, CaCO_3_ precipitated almost immediately in the H_2_O-dominant 20 and 40 mol % systems (Figure S10e,f). Both calcite (δ_CO_3__—712 cm^–1^; γ_CO_3__—873 cm^–1^) and aragonite (γ_CO_3__—854 cm^–1^) precipitated from the 0 and 20 mol % dispersions. The lack of a definitive δ_CO_3__ vibration made it difficult to distinguish calcite from vaterite in the 40 mol % spectra. The isolated formation of aragonite in the 0 and 20 mol % systems can be attributed to the dissolution of Ca(OH)_2_ and CO_2_ in H_2_O and CH_3_OH. It has been reported that limiting CO_3_^2–^ concentration, such that the Ca^2+^ concentration is greater, lowers the supersaturation at the diffusion layer and subsequently promotes aragonite crystallization.^32^ In this study, this was achieved by the slow addition of CO_2_ (70 mL/min) to a concentrated Ca(OH)_2_ dispersion (55 g/L). Furthermore, the Ca^2+^ and CO_3_^2–^ concentrations increased and decreased respectively with increasing H_2_O content (Figure S9). Aragonite formation was prevalent in the two H_2_O-dominant systems since at near ambient temperature the Ca(OH)_2_ solubility increases from 0.1 g/L in CH_3_OH to 1.65 g/L in H_2_O,^20,99^ but CO_2_ solubility decreases from 3.84 mL/ml in CH_3_OH to 0.83 mL/ml in H_2_O.^100^

Calcite precipitation was evident in all systems except the 100 mol % CH_3_OH, where the CaCO_3_ formed after 45 min of carbonation was undetectable *in situ*. However, the post-synthesis *ex situ* mid-IR showed traces of CaCO_3_ (γ_CO_3__—873 cm^–1^), much like the spectrum in Figure 2a. Figure 12b clearly shows that the precipitation rate increased with H_2_O composition. Ca(OH)_2_ completely converted into calcite within 15 min of carbonation in the pure H_2_O system. Comparatively, about 86% less of calcite was detected at this time interval in the presence of 20 mol % CH_3_OH. The expected increase in H_2_O/H_2_CO_3_, which promotes both the Ca(OH)_2_ methoxylation (Reactions 5 and 6) and Ca(OCOOCH_3_)_2_ hydrolysis (Reaction 8), was reflected in the 20 and 40 mol % by an increase in calcite precipitation after the initial 15 min. Conclusively, Figures S10–S12 confirm that the rate of CaCO_3_ formation from methanolic Ca(OH)_2_ dispersions is highly dependent on the presence of H_2_O.

## Conclusions

Different CaCO_3_ polymorphs were formed by carbonation of methanolic Ca(OH)_2_ dispersions, with CH_3_OH content varying from 0 to 100 mol %. Combined *ex situ* mid-IR, TGA, XRD, XAS and SEM confirmed that ACC, vaterite and calcite formed via transient Ca(OH)(OCH_3_), Ca(OCH_3_)_2_ and Ca(OCOOCH_3_)_2_ species in the 100 mol % CH_3_OH system. Traces of aragonite were also detected in the mid-IR. CaCO_3_ formed by hydrolysis or thermal decomposition of the Ca(OCOOCH_3_)_2_ carbonate ester. Addition of H_2_O increased both the precipitation and hydrolysis of the ester. Diffusion-limited CaCO_3_ transformations were promoted by the formation of an ACC sol–gel in the presence of ≤20 mol %H_2_O. Time-resolved XRD analysis, of both the 90 and 100 mol % systems, determined that the Ca(OCOOCH_3_)_2_ hydrolyzed into ACC, which subsequently crystallized into vaterite and then calcite. *In situ* mid-IR showed that calcite formed almost immediately in H_2_O-dominated (≥50 mol %) systems. Furthermore, the rod-like morphology of the Ca(OCOOCH_3_)_2_ carbonate ester was confirmed, but its short- to long-range structure could not be unequivocally identified. Mid-IR, XRD and XAS structural analysis highlighted the possibility of carbonic acid ester polymorphism and/or hydration. Confirmation of polymorphs and/or hydrates will require further structural analysis on a pure Ca(OCOOCH_3_)_2_. Overall, the results show that four different CaCO_3_ polymorphs can be formed in methanolic Ca(OH)_2_ dispersions. The prevalence of each polymorph is highly dependent on the CH_3_OH to H_2_O ratio and the associated formation of a sol–gel.

## Data Availability

All data supporting this study are provided either in the results section of this paper or in the electronic supplementary information accompanying it.

## Supporting Information Available

Figures of the sol–gel product, full deconvoluted mid-IR spectrum of Ca(OCOOCH_3_)_2_, possible Ca(OCOOCH_3_)_2_ conformers, complementary mid-IR spectra of gases evolved during TGA, time-resolved XRD showing the transformation of Ca(OCOOCH_3_)_2_ to CaCO_3_, reference XANES spectra of calcite, aragonite and vaterite, EDX spectra of the ester and sol–gel, inductively coupled plasma-optical emission spectrometry data of Ca(OH)_2_ dissolved in CH_3_OH/H_2_O systems. Tables of mid-IR vibration assignments for Ca(OCOOCH_3_)_2_ and the time-resolved systems also included (PDF)

## References

[ref1] Meldrum F. C. Calcium carbonate in biomineralisation and biomimetic chemistry. Int. Mater. Rev. 2003, 48 (3), 187–224. 10.1179/095066003225005836.

[ref2] Meldrum F. C.; Colfen H. Controlling mineral morphologies and structures in biological and synthetic systems. Chem. Rev. 2008, 108 (11), 4332–4432. 10.1021/cr8002856. 19006397

[ref3] Addadi L.; Raz S.; Weiner S. Taking advantage of disorder: amorphous calcium carbonate and its roles in biomineralization. Adv. Mater. 2003, 15 (12), 959–970. 10.1002/adma.200300381.

[ref4] Yasue T.; Mamiya A.; Takahashi Y.; Tsukisaka R.; Arai Y. Synthesis and characteristics of amorphous calcium carbonate. Nippon Kagaku Kaishi 1984, 1984 (7), 1107–1113. 10.1246/nikkashi.1984.1107.

[ref5] Yasue T.; Mamiya A.; Fukushima T.; Arai Y. Synthesis and characteristics of amorphous calcium carbonate in ethanol. Gypsum Lime 1985, 1985 (198), 245–252. 10.11451/mukimate1953.1985.245.

[ref6] Ueda Y.; Komatu K.; Shimizu S.; Nishioka H.; Hanazaki M.; Minayoshi S. Formation and coagulation processes of vaterite in the reaction of the system Ca (OH)_2_-CH_3_OH-H_2_O-CO_2_. Gypsum Lime 1994, 1994 (249), 105–114. 10.11451/mukimate1953.1994.105.

[ref7] Manoli F.; Dalas E. Spontaneous precipitation of calcium carbonate in the presence of ethanol, isopropanol and diethylene glycol. J. Cryst. Growth 2000, 218 (2–4), 359–364. 10.1016/S0022-0248(00)00560-1.

[ref8] Dickinson S. R.; McGrath K. M. Switching between kinetic and thermodynamic control: calcium carbonate growth in the presence of a simple alcohol. J. Mater. Chem. 2003, 13 (4), 928–933. 10.1039/b208741n.

[ref9] Park J.-K.; Ahn J.-W.; Park Y.-S.; Han C. Characteristic of crystal transition of amorphous calcium carbonate in H_2_O, ethyl and propyl alcohol system. Geosyst. Eng. 2004, 7 (4), 89–94. 10.1080/12269328.2004.10541226.

[ref10] Seo K. S.; Han C.; Wee J. H.; Park J. K.; Ahn J. W. Synthesis of calcium carbonate in a pure ethanol and aqueous ethanol solution as the solvent. J. Cryst. Growth 2005, 276 (3–4), 680–687. 10.1016/j.jcrysgro.2004.11.416.

[ref11] Lee H. S.; Ha T. H.; Kim K. Fabrication of unusually stable amorphous calcium carbonate in an ethanol medium. Mater. Chem. Phys. 2005, 93 (2–3), 376–382. 10.1016/j.matchemphys.2005.03.037.

[ref12] Chen S. F.; Yu S. H.; Jiang J.; Li F. Q.; Liu Y. K. Polymorph discrimination of CaCO_3_ mineral in an ethanol/water solution: Formation of complex vaterite superstructures and aragonite rods. Chem. Mater. 2006, 18 (1), 115–122. 10.1021/cm0519028.

[ref13] Sand K. K.; Rodriguez-Blanco J. D.; Makovicky E.; Benning L. G.; Stipp S. L. S. Crystallization of CaCO_3_ in water-alcohol mixtures: spherulitic growth, polymorph stabilization, and morphology change. Cryst. Growth Des. 2012, 12 (2), 842–853. 10.1021/cg2012342.

[ref14] Chen S. F.; Colfen H.; Antonietti M.; Yu S. H. Ethanol assisted synthesis of pure and stable amorphous calcium carbonate nanoparticles. Chem. Commun. 2013, 49 (83), 9564–9566. 10.1039/c3cc45427d. 24022058

[ref15] Rodriguez-Navarro C.; Suzuki A.; Ruiz-Agudo E. Alcohol dispersions of calcium hydroxide nanoparticles for stone conservation. Langmuir 2013, 29 (36), 11457–11470. 10.1021/la4017728. 23919634

[ref16] Rodriguez-Navarro C.; Elert K.; Sevcik R. Amorphous and crystalline calcium carbonate phases during carbonation of nanolimes: implications in heritage conservation. CrystEngComm 2016, 18 (35), 6594–6607. 10.1039/C6CE01202G.

[ref17] Rodriguez-Navarro C.; Vettori I.; Ruiz-Agudo E. Kinetics and mechanism of calcium hydroxide conversion into calcium alkoxides: implications in heritage conservation using nanolimes. Langmuir 2016, 32 (20), 5183–5194. 10.1021/acs.langmuir.6b01065. 27149182

[ref18] Hu Y. D.; Zhou Y. H.; Xu X. R.; Tang R. K. Phase-controlled crystallization of amorphous calcium carbonate in ethanol-water binary solvents. Cryst. Res. Technol. 2015, 50 (4), 312–318. 10.1002/crat.201400470.

[ref19] Magnabosco G.; Polishchuk I.; Pokroy B.; Rosenberg R.; Colfen H.; Falini G. Synthesis of calcium carbonate in trace water environments. Chem. Commun. 2017, 53 (35), 4811–4814. 10.1039/C7CC01342F. 28417115

[ref20] Gryglewicz S. Alkaline-earth metal compounds as alcoholysis catalysts for ester oils synthesis. Appl. Catal., A 2000, 192 (1), 23–28. 10.1016/S0926-860X(99)00337-3.

[ref21] Day R. L. Reactions between methanol and portland-cement paste. Cem. Concr. Res. 1981, 11 (3), 341–349. 10.1016/0008-8846(81)90106-X.

[ref22] Beaudoin J. J. Validity of using methanol for studying the microstructure of cement paste. Mater. Struct. 1987, 20 (1), 27–31. 10.1007/BF02472723.

[ref23] Beaudoin J. J.; Gu P.; Marchand J.; Tamtsia B.; Myers R. E.; Liu Z. Solvent replacement studies of hydrated portland cement systems: The role of calcium hydroxide. Adv. Cem. Based Mater. 1998, 8 (2), 56–65. 10.1016/S1065-7355(98)00008-X.

[ref24] Withum J. A.; Yoon H. Y. Treatment of hydrated lime with methanol for in-duct desulfurization sorbent improvement. Environ. Sci. Technol. 1989, 23 (7), 821–827. 10.1021/es00065a010.

[ref25] Buzágh A. Ueber kolloide lösungen der erdalkalikarbonate. Kolloid-Z. 1926, 38 (3), 222–226. 10.1007/BF01460833.

[ref26] Plank J.; Hoffmann H.; Schlkopf J.; Seidl W.; Zeitler I.; Zhang Z. Preparation and characterization of a calcium carbonate aerogel. Res. Lett. Mater. Sci. 2009, 2009, 1–3. 10.1155/2009/138476.

[ref27] Witkamp G. J.; Escobar S. A. P.; Gaertner R. S. Method for producing calcium carbonate gel and product obtained thereby. U.S. Patent 20,150,344,319 A1, 2015.

[ref28] Berner E. Über die Einwirkung der Erdalkalioxyde auf Alkohole. Ber. Dtsch. Chem. Ges. 1938, 71, 2015–2021. 10.1002/cber.19380710938.

[ref29] Grigor’ev A. I.; Turova N. Y. Infrared absorption spectra of alcoholates of beryllium magnesium and alkali earth metals. Dokl. Akad. Nauk SSSR 1965, 162 (1), 98–101.

[ref30] Kubo T.; Uchida K.; Tsubosaki K.; Hashimi F. Studies of reactions between metal hydroxides and alcohols.2. reactions between CDI_2_ structured metal (ii) hydroxides and CH_3_OH. Kogyo Kagaku Zasshi 1970, 73 (1), 75–82. 10.1246/nikkashi1898.73.75.

[ref31] Arai Y.; Yasue T.; Wakui Y. Methoxidation of calcium hydroxide and characteristics of its compound. Nippon Kagaku Kaishi 1981, 1981 (9), 1402–1408. 10.1246/nikkashi.1981.1402.

[ref32] Kitamura M.; Konno H.; Yasui A.; Masuoka H. Controlling factors and mechanism of reactive crystallization of calcium carbonate polymorphs from calcium hydroxide suspensions. J. Cryst. Growth 2002, 236 (1–3), 323–332. 10.1016/S0022-0248(01)02082-6.

[ref33] Shivkumara C.; Singh P.; Gupta A.; Hegde M. S. Synthesis of vaterite CaCO_3_ by direct precipitation using glycine and L-alanine as directing agents. Mater. Res. Bull. 2006, 41 (8), 1455–1460. 10.1016/j.materresbull.2006.01.026.

[ref34] Koga N.; Nakagoe Y. Z.; Tanaka H. Crystallization of amorphous calcium carbonate. Thermochim. Acta 1998, 318 (1–2), 239–244. 10.1016/S0040-6031(98)00348-7.

[ref35] Wojdyr M. Fityk: a general-purpose peak fitting program. J. Appl. Crystallogr. 2010, 43 (5), 1126–1128. 10.1107/S0021889810030499.

[ref36] Degen T.; Sadki M.; Bron E.; Konig U.; Nenert G. The HighScore suite. Powder Diffr. 2014, 29, S13–S18. 10.1017/S0885715614000840.

[ref37] Dent A. J.; Cibin G.; Ramos S.; Smith A. D.; Scott S. M.; Varandas L.; Pearson M. R.; Krumpa N. A.; Jones C. P.; Robbins P. E. B18: A core XAS spectroscopy beamline for Diamond. In 14th International Conference on X-Ray Absorption Fine Structure (XAFS14), Iop Publishing Ltd; Camerino, ITALY, 2009; 190, 012039. 10.1088/1742-6596/190/1/012039.

[ref38] Ravel B.; Newville M. Athena, Artemis, Hephaestus: data analysis for X-ray absorption spectroscopy using IFEFFIT. J. Synchrotron Radiat. 2005, 12, 537–541. 10.1107/S0909049505012719. 15968136

[ref39] Effenberger H. Crystal structure and infrared-absorption spectrum of synthetic monohydrocalcite, CaCO_3_.H_2_O. Monatsh. Chem. 1981, 112 (8–9), 899–909. 10.1007/BF00905061.

[ref40] Demichelis R.; Raiteri P.; Gale J. D.; Dovesi R. The multiple structures of vaterite. Cryst. Growth Des. 2013, 13 (6), 2247–2251. 10.1021/cg4002972.

[ref41] Lutz H. D. Zur kenntnis der erdalkalimethylate -IR-spektroskopische und rontgenographische untersuchungen an Mg(OCH_3_)_2_ Ca(OCH_3_)_2_ Sr(OCH_3_)_2_ und Ba(OCH_3_)_2_. Z. Anorg. Allg. Chem. 1967, 353 (3–4), 207–215. 10.1002/zaac.19673530312.

[ref42] Andersen F. A.; Brecevic L.; Beuter G.; Dell’Amico D. B.; Calderazzo F.; Bjerrum N. J.; Underhill A. E. Infrared spectra of amorphous and crystalline calcium carbonate. Acta Chem. Scand. 1991, 45 (10), 1018–1024. 10.3891/acta.chem.scand.45-1018.

[ref43] Gebauer D.; Gunawidjaja P. N.; Ko J. Y. P.; Bacsik Z.; Aziz B.; Liu L. J.; Hu Y. F.; Bergstrom L.; Tai C. W.; Sham T. K.; et al. Proto-calcite and proto-vaterite in amorphous calcium carbonates. Angew. Chem., Int. Ed. 2010, 49 (47), 8889–8891. 10.1002/anie.201003220. 20949576

[ref44] Tao J. Chapter twenty-two-FTIR and Raman studies of structure and bonding in mineral and organic-mineral composites. In Methods in Enzymology; Yoreo J. D., Ed.; Academic Press, 2013; Vol. 532, pp 533–556. 10.1016/B978-0-12-416617-2.00022-9 24188781

[ref45] Ito K.; Bernstein H. J. The vibrational spectra of the formate, acetate, and oxalate ions. Can. J. Chem. 1956, 34 (2), 170–178. 10.1139/v56-021.

[ref46] Musumeci A. W.; Frost R. L.; Waclawik E. R. A spectroscopic study of the mineral paceite (calcium acetate). Spectrochim. Acta, Part A 2007, 67 (3–4), 649–661. 10.1016/j.saa.2006.07.045. 17070100

[ref47] Valor A.; Reguera E.; Sánchez-Sinencio F. Synthesis and X-ray diffraction study of calcium salts of some carboxylic acids. Powder Diffr. 2002, 17 (1), 13–18. 10.1154/1.1414011.

[ref48] Mattes R.; Scholten K. Vibrational-spectra and force constants in monoalkylcarbonates and monoalkylthiocarbonates. Spectrochim. Acta, Part A 1975, 31 (9–10), 1307–1315. 10.1016/0584-8539(75)80188-7.

[ref49] Matsuta S.; Asada T.; Kitaura K. Vibrational assignments of lithium alkyl carbonate and lithium alkoxide in the infrared spectra - An ab initio MO study. J. Electrochem. Soc. 2000, 147 (5), 1695–1702. 10.1149/1.1393420.

[ref50] Zhuang G. V.; Yang H.; Ross P. N.; Xu K.; Jow T. R. Lithium methyl carbonate as a reaction product of metallic lithium and dimethyl carbonate. Electrochem. Solid-State Lett. 2006, 9 (2), A64–A68. 10.1149/1.2142157.

[ref51] Kornprobst T.; Plank J. Synthesis and properties of magnesium carbonate xerogels and aerogels. J. Non-Cryst. Solids 2013, 361, 100–105. 10.1016/j.jnoncrysol.2012.10.023.

[ref52] Katon J. E.; Cohen M. D. The vibrational spectra and structure of dimethyl carbonate and its conformational behavior. Can. J. Chem. 1975, 53 (9), 1378–1386. 10.1139/v75-191.

[ref53] Lang P. L.; Katon J. E. The vibrational-spectra, structure, and conformational behavior of dimethyl dicarbonate. J. Mol. Struct. 1988, 172, 113–128. 10.1016/0022-2860(88)87010-8.

[ref54] Roeges N. P. G. A Guide to the Complete Interpretation of Infrared Spectral of Organic Structures; John Wiley & Sons Ltd., 1994.

[ref55] Behrens G.; Kuhn L. T.; Ubic R.; Heuer A. H. Raman spectra of vateritic calcium carbonate. Spectrosc. Lett. 1995, 28 (6), 983–995. 10.1080/00387019508009934.

[ref56] Pilati T.; Demartin F.; Gramaccioli C. M. Lattice-dynamical estimation of atomic displacement parameters in carbonates: Calcite and aragonite CaCO_3_, dolomite CaMg(CO_3_)_2_ and magnesite MgCO_3_. Acta Crystallogr., Sect. B: Struct. Sci., Cryst. Eng. Mater. 1998, 54, 515–523. 10.1107/S0108768197018181.

[ref57] Wang J. W.; Becker U. Structure and carbonate orientation of vaterite (CaCO_3_). Am. Mineral. 2009, 94 (2–3), 380–386. 10.2138/am.2009.2939.

[ref58] Bhagavantam S.; Venkatarayudu T. Raman effect in relation to crystal structure. Proc. Natl. Acad. Sci. 1939, 9 (3), 224–258. 10.1007/BF03046465.

[ref59] Herzberg G. Molecular Spectra and Molecular Structure. II. Infrared and Raman Spectra of Polyatomic Molecules; Van Nostrand, 1939.

[ref60] Ryskin Y. I. The vibrations of protons in minerals: hydroxyl, water and ammonium. In The Infrared Spectra of Minerals; Farmer V. C., Ed.; Mineralogical Society of Great Britain and Ireland, 1974.

[ref61] Teo S. H.; Taufiq-Yap Y. H.; Rashid U.; Islam A. Hydrothermal effect on synthesis, characterization and catalytic properties of calcium methoxide for biodiesel production from crude Jatropha curcas. RSC Adv. 2015, 5 (6), 4266–4276. 10.1039/C4RA11936C.

[ref62] Masood H.; Yunus R.; Choong T. S. Y.; Rashid U.; Taufiq Yap Y. H. Synthesis and characterization of calcium methoxide as heterogeneous catalyst for trimethylolpropane esters conversion reaction. Appl. Catal., A 2012, 425–426, 184–190. 10.1016/j.apcata.2012.03.019.

[ref63] Cross J.; Hunter R.; Stimson V. The thermal decomposition of simple carbonate esters. Aust. J. Chem. 1976, 29 (7), 1477–1481. 10.1071/CH9761477.

[ref64] Staeglich H.; Weiss E. Crystal structures of alkaline-earth methanolates M(OCH_3_)_2_,M = Ca,Sr,Ba. Chem. Ber./Recl. 1978, 111 (3), 901–905. 10.1002/cber.19781110310.

[ref65] Markgraf S. A.; Reeder R. J. High-temperature structure refinements of calcite and magnesite. Am. Mineral. 1985, 70 (5–6), 590–600.

[ref66] Radha A. V.; Forbes T. Z.; Killian C. E.; Gilbert P.; Navrotsky A. Transformation and crystallization energetics of synthetic and biogenic amorphous calcium carbonate. Proc. Natl. Acad. Sci. U.S.A. 2010, 107 (38), 16438–16443. 10.1073/pnas.1009959107. 20810918 PMC2944757

[ref67] Ma Y. F.; Feng Q. L. A crucial process: organic matrix and magnesium ion control of amorphous calcium carbonate crystallization on β-chitin film. CrystEngComm 2015, 17 (1), 32–39. 10.1039/C4CE01616E.

[ref68] Ihli J.; Wong W. C.; Noel E. H.; Kim Y. Y.; Kulak A. N.; Christenson H. K.; Duer M. J.; Meldrum F. C. Dehydration and crystallization of amorphous calcium carbonate in solution and in air. Nat. Commun. 2014, 5, 3169 10.1038/ncomms4169. 24469266 PMC4085778

[ref69] Kan A. T.; Fu G. M.; Tomson M. B. Effect of methanol on carbonate equilibrium and calcite solubility in a gas/methanol/water/salt mixed system. Langmuir 2002, 18 (25), 9713–9725. 10.1021/la025620n.

[ref70] Fulton J. L.; Heald S. M.; Badyal Y. S.; Simonson J. M. Understanding the effects of concentration on the solvation structure of Ca^2+^ in aqueous solution. I: The perspective on local structure from EXAFS and XANES. J. Phys. Chem. A 2003, 107 (23), 4688–4696. 10.1021/jp0272264.

[ref71] Wolf S. E.; Muller L.; Barrea R.; Kampf C. J.; Leiterer J.; Panne U.; Hoffmann T.; Emmerling F.; Tremel W. Carbonate-coordinated metal complexes precede the formation of liquid amorphous mineral emulsions of divalent metal carbonates. Nanoscale 2011, 3 (3), 1158–1165. 10.1039/c0nr00761g. 21218241 PMC3111071

[ref72] Cabaret D.; Emery N.; Bellin C.; Herold C.; Lagrange P.; Wilhelm F.; Rogalev A.; Loupias G. Nature of empty states in superconducting CaC_6_ and related Li-Ca ternary graphite intercalation compounds using polarized x-ray absorption near-edge structure at the Ca K edge. Phys. Rev. B 2013, 87 (7), 075108 10.1103/physrevb.87.075108.

[ref73] Henderson G. S.; de Groot F. M. F.; Moulton B. J. A. X-ray Absorption Near-Edge Structure (XANES) Spectroscopy. Rev. Mineral. Geochem. 2014, 78, 75–138. 10.2138/rmg.2014.78.3.

[ref74] Sowrey F. E.; Skipper L. J.; Pickup D. M.; Drake K. O.; Lin Z.; Smith M. E.; Newport R. J. Systematic empirical analysis of calcium-oxygen coordination environment by calcium K-edge XANES. Phys. Chem. Chem. Phys. 2004, 6 (1), 188–192. 10.1039/B311715D.

[ref75] Geere R. G.; Gaskell P. H.; Greaves G. N.; Greengrass J.; Binstead N. EXAFS and XANES spectra of calcium silicate glasses. In EXAFS and Near Edge Structure; Bianconi A., Incoccia L., Stipcich S., Eds.; Springer Series in Chemical Physics; Springer: Berlin Heidelberg, 1983; Vol. 27, pp 256–260. 10.1007/978-3-642-50098-5_55.

[ref76] Martin J. M.; Belin M.; Mansot J. L. EXAFS of calcium in overbased micelles. J. Phys. 1986, 47 (C8), C8-887–C8-890. 10.1051/jphyscol:19868172.

[ref77] Yamashita H.; Nomura M.; Tomita A. Local structures of metals dispersed on coal.4. local-structure of calcium species on coal after heat-treatment and co_2_ gasification. Energy Fuels 1992, 6 (5), 656–661. 10.1021/ef00035a018.

[ref78] Guo X. X.; Wu J.; Yiu Y. M.; Hu Y. F.; Zhu Y. J.; Sham T. K. Drug-nanocarrier interaction-tracking the local structure of calcium silicate upon ibuprofen loading with X-ray absorption near edge structure (XANES). Phys. Chem. Chem. Phys. 2013, 15 (36), 15033–15040. 10.1039/c3cp50699a. 23925643

[ref79] Odin G. P.; Vanmeert F.; Farges F.; Gand G.; Janssens K.; Romero-Sarmiento M. F.; Steyer J. S.; Vantelon D.; Rouchon V. Alteration of fossil-bearing shale (Autun, France; Permian), part II: Monitoring artificial and natural ageing by combined use of S and Ca K-edge XANES analysis, Rock-Eval pyrolysis and FTIR analysis. Ann. Paleontol. 2015, 101 (3), 225–239. 10.1016/j.annpal.2015.03.001.

[ref80] Newville M. Fundamentals of XAFS. Rev. Mineral. Geochem. 2014, 78, 33–74. 10.2138/rmg.2014.78.2.

[ref81] Gunther C.; Becker A.; Wolf G.; Epple M. In vitro synthesis and structural characterization of amorphous calcium carbonate. Z. Anorg. Allg. Chem. 2005, 631 (13–14), 2830–2835. 10.1002/zaac.200500164.

[ref82] Levi-Kalisman Y.; Raz S.; Weiner S.; Addadi L.; Sagi I. X-Ray absorption spectroscopy studies on the structure of a biogenic “amorphous” calcium carbonate phase. J. Chem. Soc., Dalton Trans. 2000, 2000 (21), 3977–3982. 10.1039/b003242p.

[ref83] Politi Y.; Levi-Kalisman Y.; Raz S.; Wilt F.; Addadi L.; Weiner S.; Sagi I. Structural characterization of the transient amorphous calcium carbonate precursor phase in sea urchin embryos. Adv. Funct. Mater. 2006, 16 (10), 1289–1298. 10.1002/adfm.200600134.

[ref84] Lam R. S. K.; Charnock J. M.; Lennie A.; Meldrum F. C. Synthesis-dependant structural variations in amorphous calcium carbonate. CrystEngComm 2007, 9 (12), 1226–1236. 10.1039/b710895h.

[ref85] Taylor M. G.; Simkiss K.; Greaves G. N.; Okazaki M.; Mann S. An X-ray absorption spectroscopy study of the structure and transformation of amorphous calcium carbonate from plant cystoliths. Proc. R. Soc. B 1993, 252 (1333), 75–80. 10.1098/rspb.1993.0048.

[ref86] Becker A.; Bismayer U.; Epple M.; Fabritius H.; Hasse B.; Shi J. M.; Ziegler A. Structural characterisation of X-ray amorphous calcium carbonate (ACC) in sternal deposits of the crustacea Porcellio scaber. Dalton Trans. 2003, 2003 (4), 551–555. 10.1039/b210529b.

[ref87] Klaithong S.; Opdenbosch D. V.; Zollfrank C.; Plank J. Preparation of CaCO_3_ and CaO replicas retaining the hierarchical structure of spruce wood. Z. fur Naturforsch.—B J. Chem. Sci. 2013, 68 (5–6), 533–538. 10.5560/znb.2013-3062.

[ref88] Mugnaioli E.; Andrusenko I.; Schuler T.; Loges N.; Dinnebier R. E.; Panthofer M.; Tremel W.; Kolb U. Ab initio structure determination of vaterite by automated electron diffraction. Angew. Chem., Int. Ed. 2012, 51 (28), 7041–7045. 10.1002/anie.201200845. 22685061

[ref89] Oaki Y.; Imai H. Experimental demonstration for the morphological evolution of crystals grown in gel media. Cryst. Growth Des. 2003, 3 (5), 711–716. 10.1021/cg034053e.

[ref90] Dedonder-Lardeux C.; Gregoire G.; Jouvet C.; Martrenchard S.; Solgadi D. Charge separation in molecular clusters: Dissolution of a salt in a salt-(solvent)(n) cluster. Chem. Rev. 2000, 100 (11), 4023–4037. 10.1021/cr990059s. 11749338

[ref91] Danks A. E.; Hall S. R.; Schnepp Z. The evolution of ’sol-gel’ chemistry as a technique for materials synthesis. Mater. Horiz. 2016, 3 (2), 91–112. 10.1039/C5MH00260E.

[ref92] Cushing B. L.; Kolesnichenko V. L.; O’Connor C. J. Recent advances in the liquid-phase syntheses of inorganic nanoparticles. Chem. Rev. 2004, 104 (9), 3893–3946. 10.1021/cr030027b. 15352782

[ref93] Hench L. L.; West J. K. The sol-gel process. Chem. Rev. 1990, 90 (1), 33–72. 10.1021/cr00099a003.

[ref94] Brinker C. J.; Scherer G. W. Chapter 1 - Introduction. In Sol-Gel Science; Academic Press, 1990.

[ref95] Bots P.; Benning L. G.; Rodriguez-Blanco J. D.; Roncal-Herrero T.; Shaw S. Mechanistic Insights into the Crystallization of Amorphous Calcium Carbonate (ACC). Cryst. Growth Des. 2012, 12 (7), 3806–3814. 10.1021/cg300676b.

[ref96] Rodriguez-Blanco J. D.; Shaw S.; Benning L. G. The kinetics and mechanisms of amorphous calcium carbonate (ACC) crystallization to calcite, via vaterite. Nanoscale 2011, 3 (1), 265–271. 10.1039/C0NR00589D. 21069231

[ref97] Nielsen M. H.; Aloni S.; De Yoreo J. J. In situ TEM imaging of CaCO3 nucleation reveals coexistence of direct and indirect pathways. Science 2014, 345 (6201), 1158–1162. 10.1126/science.1254051. 25190792

[ref98] Yu L. X.; Lionberger R. A.; Raw A. S.; D’Costa R.; Wu H. Q.; Hussain A. S. Applications of process analytical technology to crystallization processes. Adv. Drug Delivery Rev. 2004, 56 (3), 349–369. 10.1016/j.addr.2003.10.012. 14962586

[ref99] Seidell A. Solubilities of Inorganic and Organic Compounds: A Compilation of Solubility Data from the Periodical Literature; D. Van Nostrand Company, 1919.

[ref100] Just G. Löslichkeit von Gasen in organischen Lösungsmitteln. Zeitsch. phy. Chem. 1901, 37U (1), 342–367. 10.1515/zpch-1901-3719.

