# Supplementary material for: Reactive CaCO3 Formation from CO2 and Methanolic Ca(OH)2 Dispersions: Transient Methoxide Salts, Carbonate Esters and Sol–Gels
Source: ACS Phys Chem Au. 2024 Jul 23;4(5):555–67. doi: 10.1021/acsphyschemau.4c00041 (PMC11447961; doi:10.1021/acsphyschemau.4c00041)
Supplement: Supplementary file 1 — pg4c00041_si_001.pdf [file pg4c00041_si_001.pdf]

Reactive $CaCO_3$ Formation from CO_2 and Methanolic $Ca(OH)_2$ Dispersions: Transient Methoxide Salts, Carbonate Esters and Sol-Gels

Thokozile A. Kathyola,[†, ‡] Elizabeth A. Willneff,[†,§] Colin J. Willis,[ǁ] Peter J. Dowding,[ǁ] and Sven L.M. Schroeder*[,†,‡]

[†]School of Chemical and Process Engineering, University of Leeds, Leeds, LS2 9JT, UK

[‡]Diamond Light Source, Harwell UK Science & Innovation Campus, Didcot, OX11 0DE, UK

[§]School of Design, University of Leeds, Leeds, LS2 9JT, UK

[ǁ]Infineum UK Ltd., Abingdon, Oxfordshire, OX13 6BB, UK

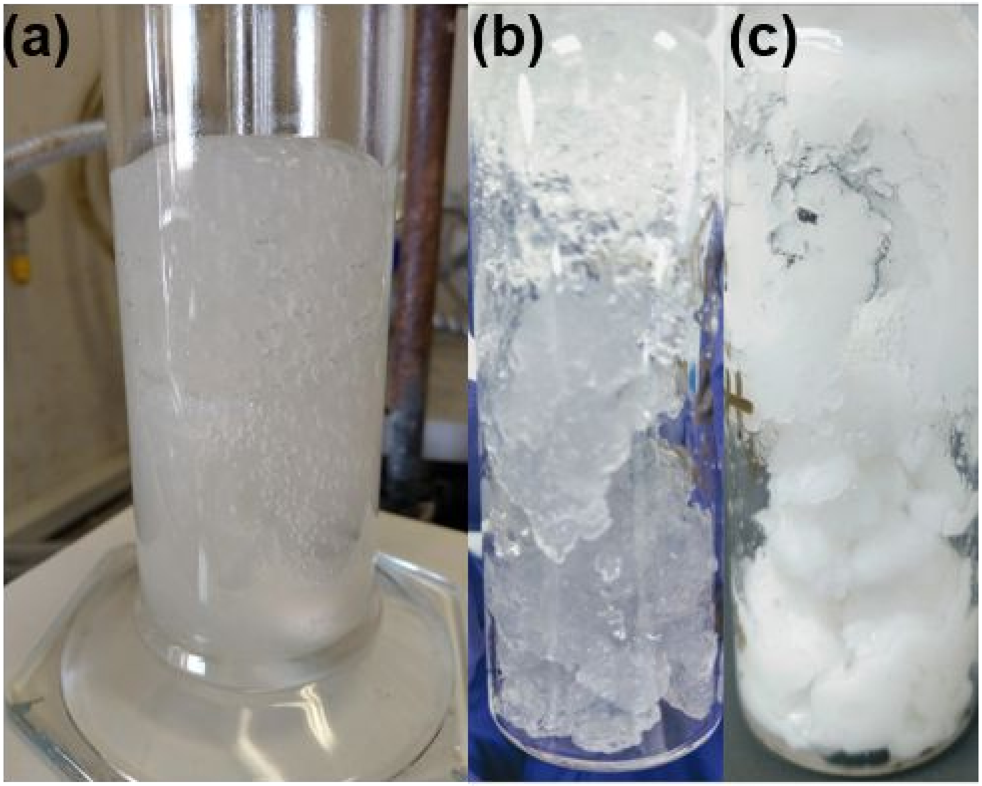

Figure S1. The product from the carbonation of $Ca(OH)_2$ in 90 mol% CH_3OH. Pictures show (a and b) an initial (<5 min) translucent sol-gel, which converts to (c) a sol-gel/white precipitate mixture (~1 h) following hydrolysis.

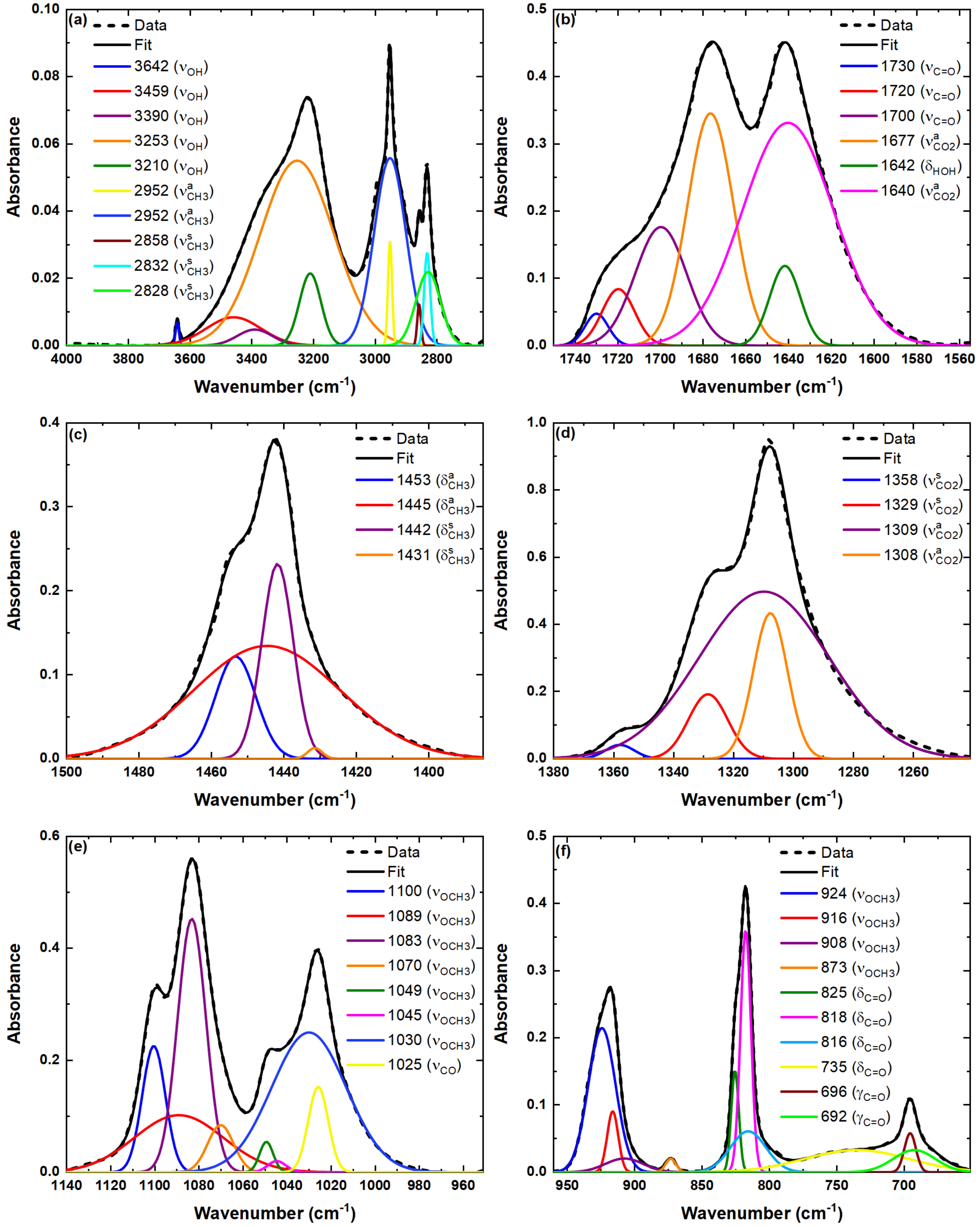

Figure S2. Deconvoluted Mid-IR plots for the initial (15 minutes) post-carbonation product from the pure (100 mol%) methanolic dispersion from 4000 to 650 cm^{-1} (a to f). Vibrations due to methanol (CH_3OH), calcium methoxide ($Ca(OCH_3)_2$, calcium dimethyl dicarbonate ($Ca(OCOOCH_3)_2$) and water (H_2O) have been highlighted. All data is summarised in Table S1.

Table S1. Mid-IR vibrations (cm^{-1}) for the methoxycarbonyl anion ($CH_3OCO_2^-$) and the post-carbonation product from the 100 mol% methanol (M) system at 15 min (initial) and 60 h (aged). Vibrations due to calcium hydroxide (H), methoxide (MO), dimethylcarbonate (DMC) ester, calcite (C), aragonite (A) and vaterite (V) have been assigned

**Assignments**	**Anion** $CH_3OCO_2^-$ (151)	**100 mol% CH_3OH Product** Initial (15 min)		Aged (60 h)	
ν_{OH}	-	3642	H	-	-
ν_{OH}	-	3295	M or W	-	-
ν^a_{CH3}	2990 ± 40	2987; 2953; 2913	M/DMC	2926	M
ν^s_{CH3}	2920 ± 80	2893;2856	DMC/MO?	2856	DMC/MO?
$2\cdot\nu^s_{CO2}$	-	2013	DMC	-	-
$\nu^s_{CO3} + \delta_{CO3}$	-	-	-	1796	C
$\nu_{C=O}$	1750 ± 50	1730;1720; 1700	DMC	-	-
ν^a_{CO2}	-	1677; 1642	DMC	-	-
δ_{HOH}	-	1640	W	-	-
δ^a_{CH3}	1450 ± 15	1453; 1445	M/DMC	-	-
δ^s_{CH3}	1435 ± 15	1442; 1431	DMC	-	-
ν^a_{CO3}	-	-	-	1402; 1397	C
ν^s_{CO2}	-	1358	DMC	-	-
ν^s_{CO2}	-	1329	DMC	-	-
ν^a_{CO2}	1255 ± 60	1309; 1308	DMC	-	-
ρ_{CH3}	1185 ± 35	1190	DMC	-	-
ρ_{CH3}		1160	MO	-	-
ν_{OCH3}/ν^s_{CO3}		1100; 1089; 1083; 1070	DMC	1080	A
ν_{OCH3}	975 ± 125	1049; 1045; 1030	MO	-	-
ν_{CO}		1025	M	-	-
ν_{OCH3}		924; 918; 908	DMC	-	-
γ_{CO3}		873	C	873; 857; 848	C and A
$\delta_{C=O}$	715 ± 115	825; 818; 816; 735	DMC	-	-
$\gamma_{C=O}/\delta_{CO3}$	635 ± 130	696; 692	DMC	713	C

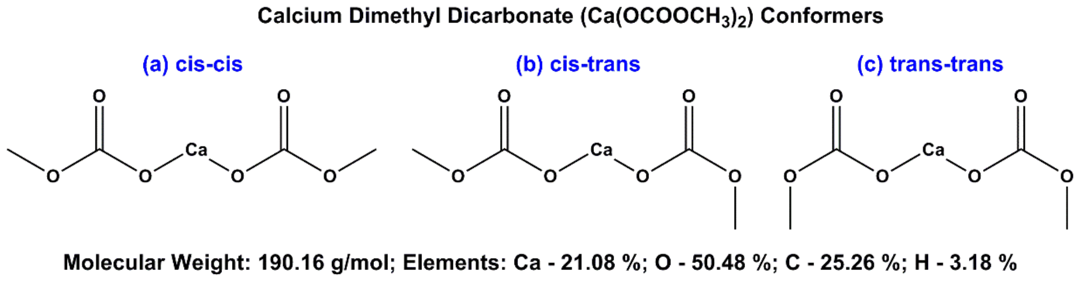

Figure S3. Possible conformations of calcium dimethyl dicarbonate ($Ca(OCOOCH_3)_2$) based on variations of the methoxy group ($-OCH_3$) orientation.

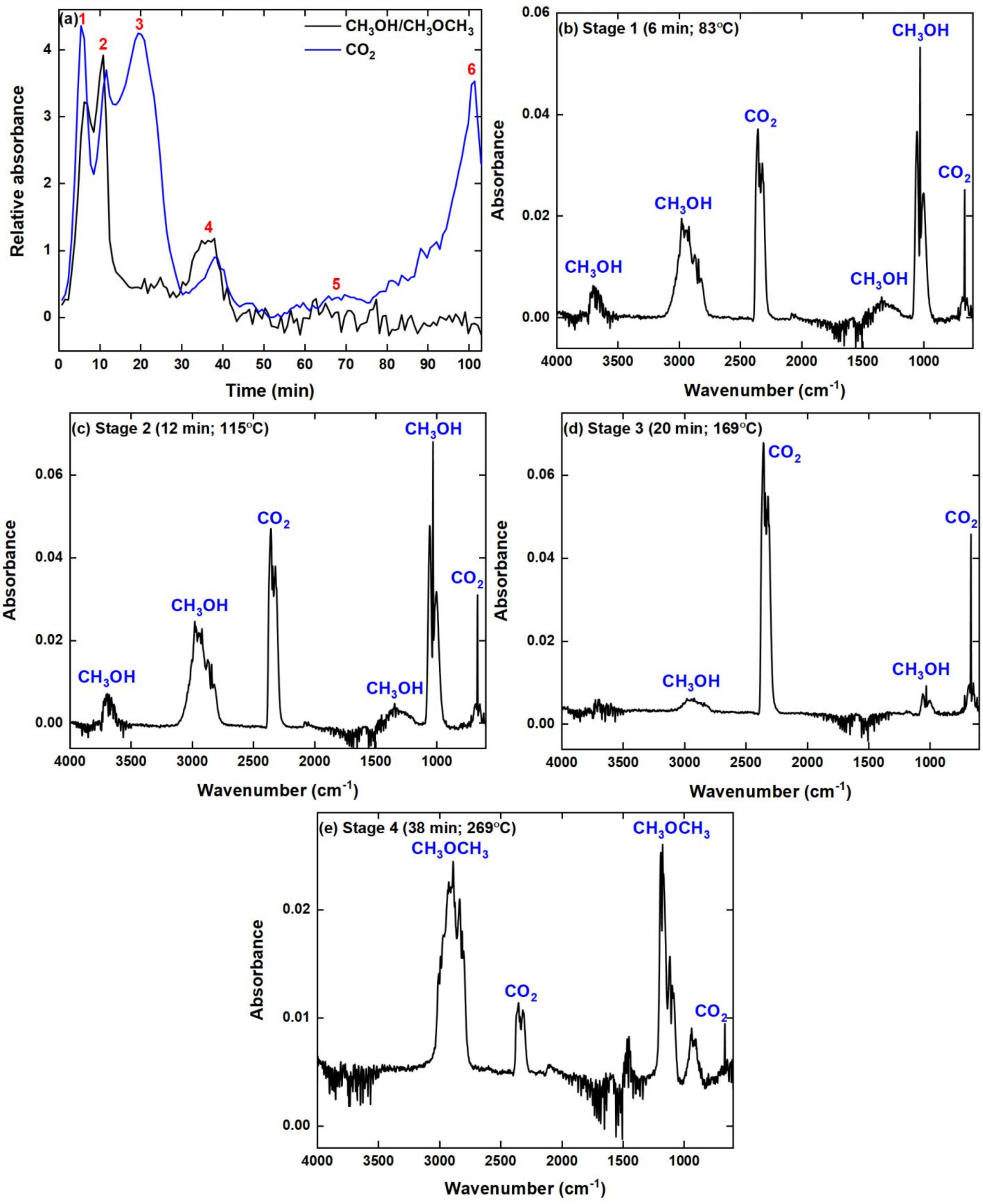

Figure S4. TGA-IR gas evolution during the thermal decomposition of $Ca(OCOOCH_3)_2$ into CO_2, CH_3OCH_3, $Ca(OCH_3)_2$ and $CaCO_3$. Residual CH_3OH was also present.

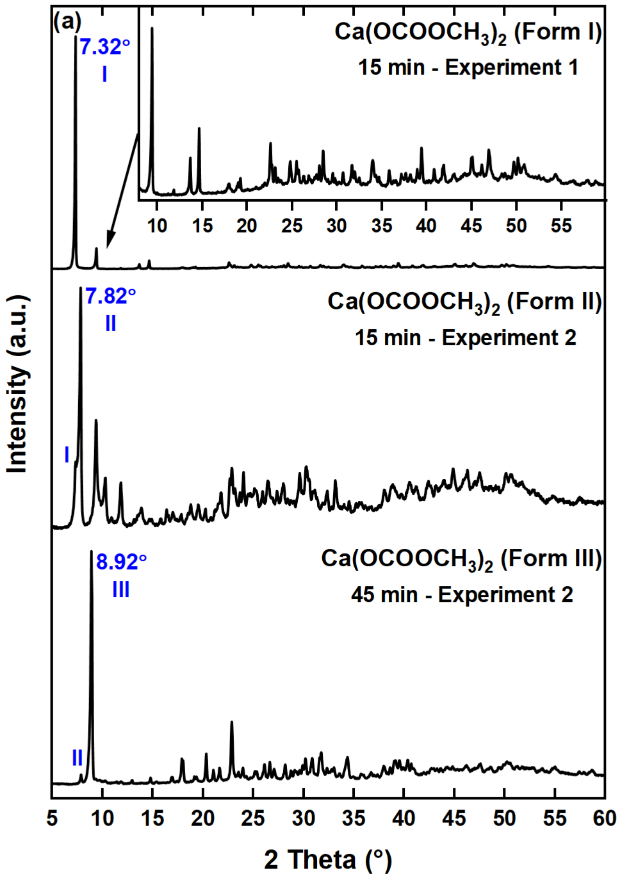

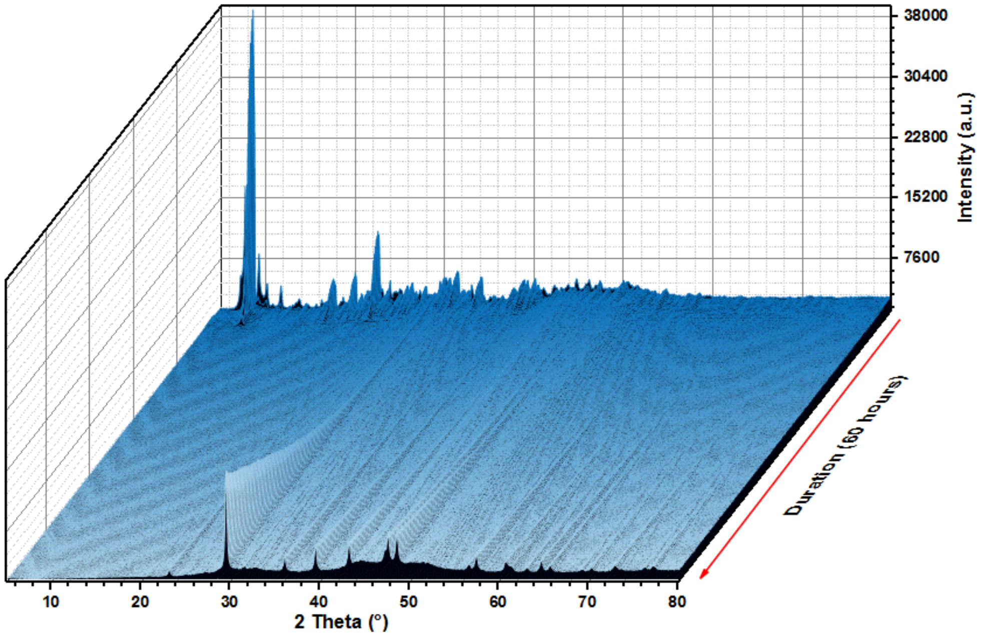

Figure S5. Time-resolved XRD plots showing the products precipitated from the carbonation of the pure (100 mol%) methanolic dispersion (experiment 2). Shows conversion from $Ca(OH)_2$ to calcite via $Ca(OCH_3)_2$, $Ca(OCOOCH_3)_2$, ACC and vaterite.

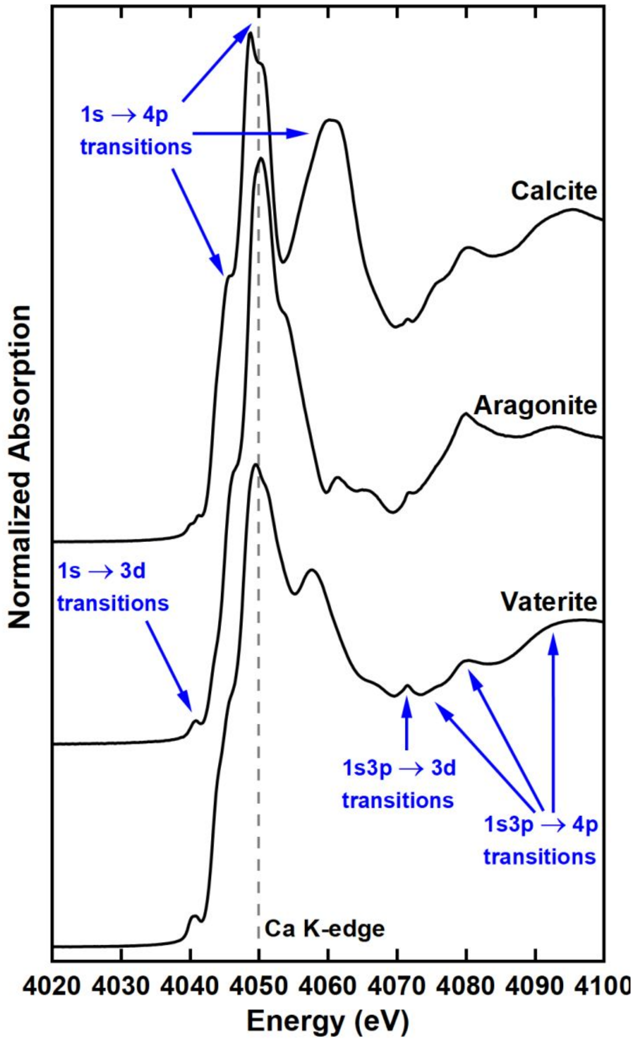

Figure S6. Ca K-edge XANES of the anhydrous $CaCO_3$ polymorphs calcite, aragonite and vaterite.

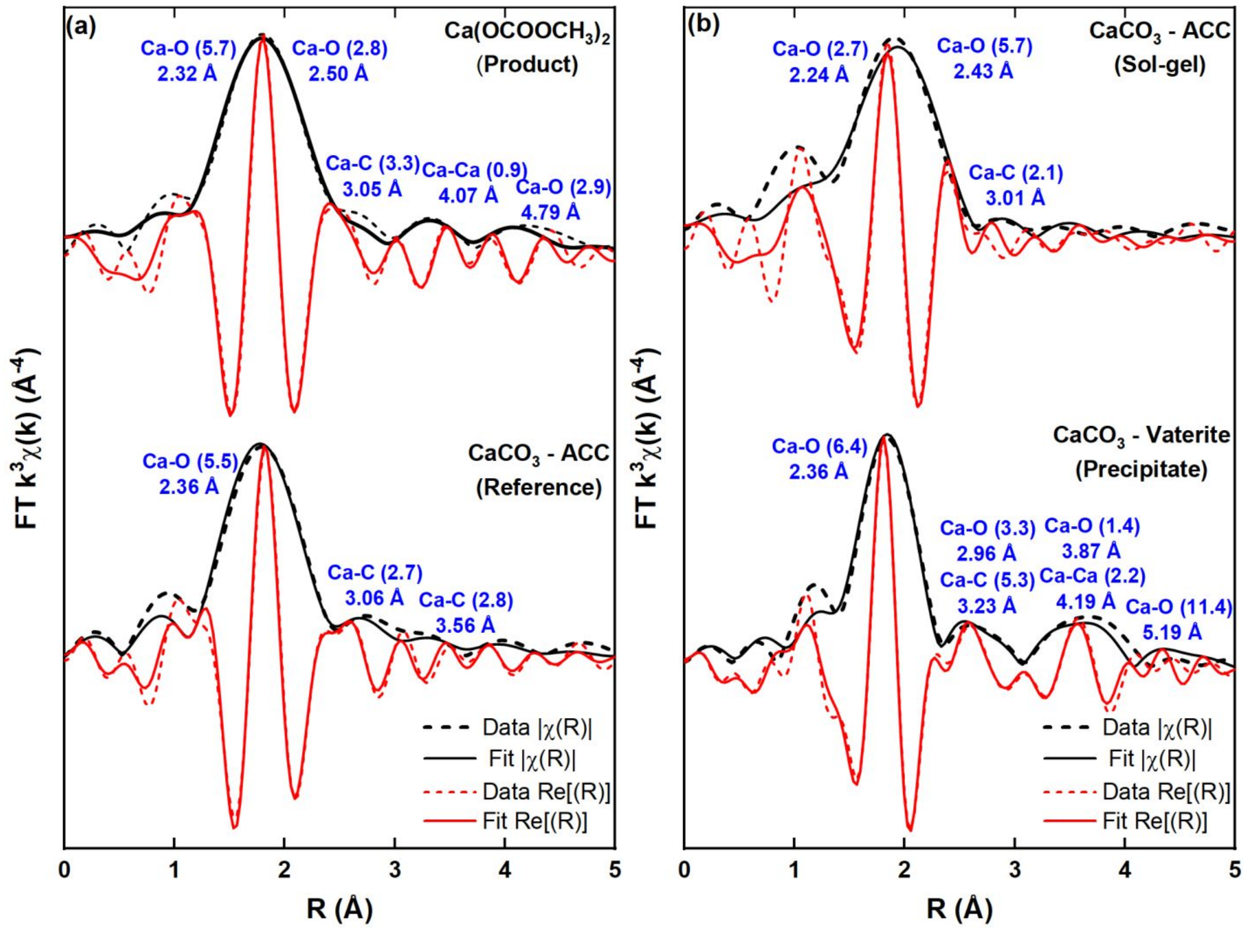

Figure S7. Ca K-edge EXAFS of (a) the initial 100 mol% CH_3OH post-carbonation product ($Ca(OCOOCH_3)_2$) compared to an ACC standard and (b) the initial (sol-gel) and aged (precipitate) 90 mol% CH_3OH post-carbonation product.

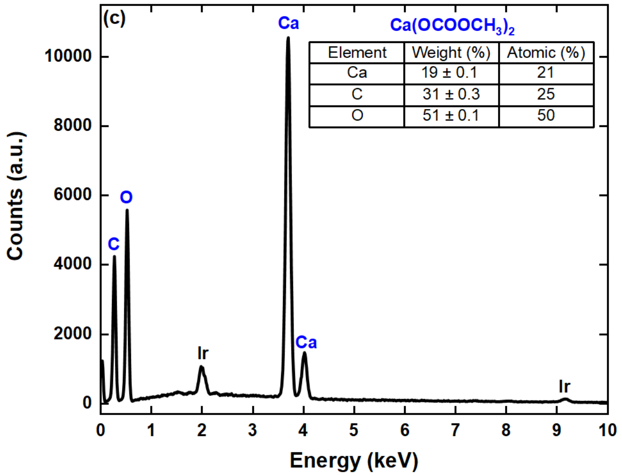

Figure S8. EDX of the $Ca(OCOOCH_3)_2$ particles synthesized from the 100 mol% methanolic $Ca(OH)_2$ dispersion.

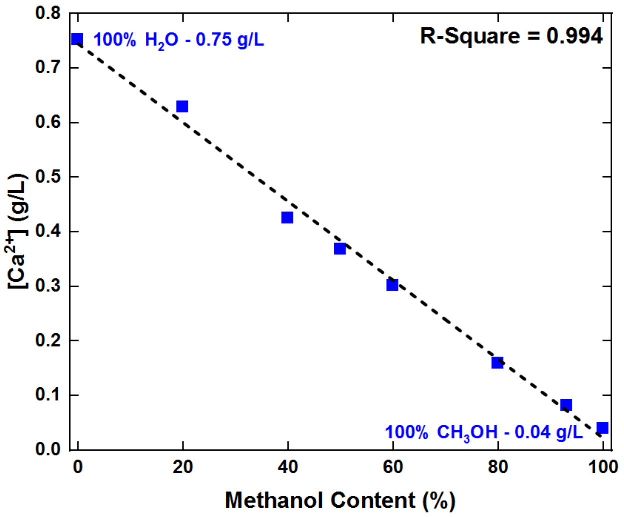

Figure S9. Concentration of Ca^{2+} ions as a function of CH_3OH content. Inductively coupled plasma-optical emission spectrometry (ICP-OES) data of $Ca(OH)_2$ dissolved in CH_3OH/H_2O systems.

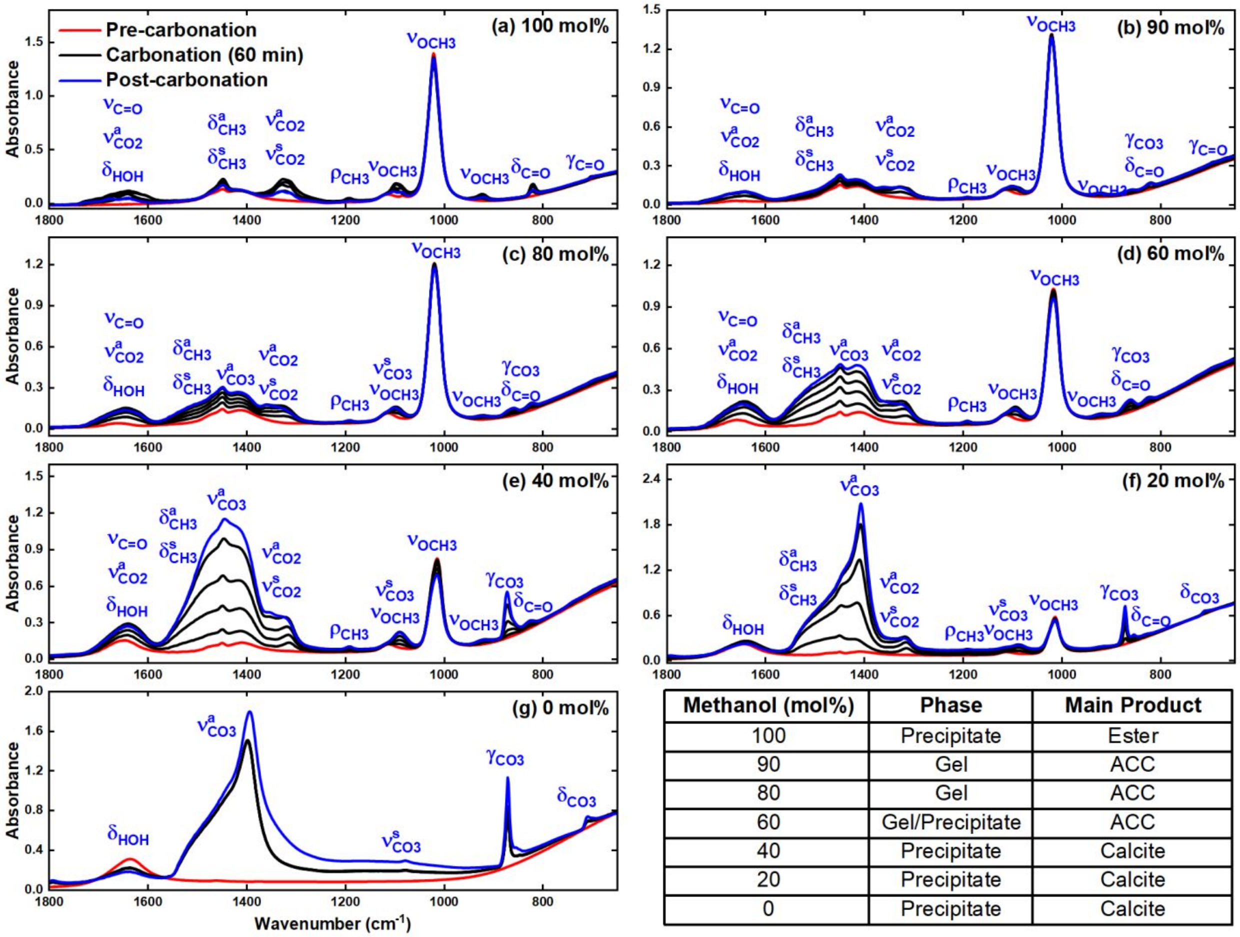

Methanol (mol%)	Phase	Main Product
100	Precipitate	Ester
90	Gel	ACC
80	Gel	ACC
60	Gel/Precipitate	ACC
40	Precipitate	Calcite
20	Precipitate	Calcite
0	Precipitate	Calcite

Figure S10. Time-resolved mid-IR spectra showing the 60-minute carbonation of seven $Ca(OH)_2$ dispersions with varying CH_3OH content (a to g –100 to 0 mol%). Each black line represents 15 min of carbonation.

Table S2. Mid-IR vibrational frequencies (cm^{-1}) for the CH_3OH, $Ca(OH)_2$, $Ca(OCOOCH_3)_2$, ACC, $CaCO_3$-α and $CaCO_3$-β involved in the carbonation of $Ca(OH)_2$ in the presence of CH_3OH (20 to 100 mol%).

**Assignments**	**Methanol Composition (mol%)**					
	**100**	**90**	**80**	**60**	**40**	**20**
ν_{OH}	3643	3643	3643	3643	3643	3643
ν_{OH}	3320	3320	3320	3320	3320	3320
$2\cdot\delta^{a}_{CH3}/\nu^{a}_{CH3}$	2982; 2944; 2915	2982; 2944; 2915	2982; 2944; 2915	2982; 2944; 2915	2982; 2953	2953
ν^{s}_{CH3}	2832	2832	2832	2832	2837	2844
$2\cdot\nu^{s}_{CO2}$	-	-	-	-	-	-
-	-	-	-	-	-	-
$\nu_{C=O}$	1721	-	-	-	-	-
ν^{a}_{CO2}	1680	-	-	-	-	-
δ_{HOH}	1643	1643	1643	1643	1643	1643
ν^{a}_{CO2}	-	-	1520	-	-	-
δ^{a}_{CH3}	1447	1448	1448	1448	1446	-
$\delta^{s}_{CH3}/\nu^{a}_{CO3}$	1412	1415	1415	1415		1408
ν^{s}_{CO2}	-	1360	1360	-	1355	-
ν^{s}_{CO2}	1330	1326	1322	1326	1322	1322
ν^{a}_{CO2}	1311	-	-	-	-	
ρ_{CH3}	1192	1192	1192	1192	1192	1190
ν_{OCH3}/ν^{s}_{CO3}	1103; 1087	1100	1100	1095	1090	1088
ν_{OCH3}	1022	1020	1020	1018	1015	-
ν_{OCH3}	918	924	924	924	924	-
ν_{OCH3}/γ_{CO3}	-	863	863	863	873	873; 854
$\delta_{C=O}$	820	825	825	825	823	830
$\gamma_{C=O}/\delta_{CO3}$	696	-	-	-	-	712
**Initial State**	**Precipitate**	**Gel**	**Gel**	**Gel/Precipitate**	**Precipitate**	**Precipitate**
